# Tumor monocyte content predicts immunochemotherapy outcomes in esophageal adenocarcinoma

**DOI:** 10.1016/j.ccell.2023.06.006

**Published:** 2023-07-10

**Authors:** Thomas M. Carroll, Joseph A. Chadwick, Richard P. Owen, Michael J. White, Joseph Kaplinsky, Iliana Peneva, Anna Frangou, Phil F. Xie, Jaeho Chang, Andrew Roth, Bob Amess, Sabrina A. James, Margarida Rei, Hannah S. Fuchs, Katy J. McCann, Ayo O. Omiyale, Brittany-Amber Jacobs, Simon R. Lord, Stewart Norris-Bulpitt, Sam T. Dobbie, Lucinda Griffiths, Kristen Aufiero Ramirez, Toni Ricciardi, Mary J. Macri, Aileen Ryan, Ralph R. Venhaus, Benoit J. Van den Eynde, Ioannis Karydis, Benjamin Schuster-Böckler, Mark R. Middleton, Xin Lu, for the LUD2015-005 Project Team, David Ahern, Bob Amess, Kristen Aufiero Ramirez, Georgina Berridge, Thomas M. Carroll, Joseph A. Chadwick, Jaeho Chang, Jingfei Cheng, Sam T. Dobbie, Magdalena Drozdz, Roman Fischer, Anna Frangou, Hannah S. Fuchs, Lucinda Griffiths, Masato Inoue, Brittany-Amber Jacobs, Sabrina A. James, Joseph Kaplinsky, Ioannis Karydis, Benedikt M. Kessler, Simon R. Lord, Hantao Lou, Xin Lu, Mary J. Macri, Katy J. McCann, Naomi McGregor, Mark R. Middleton, Stewart Norris-Bulpitt, Ayo O. Omiyale, Richard P. Owen, Iliana Peneva, Chansavath Phetsouphanh, Margarida Rei, Toni Ricciardi, Andrew Roth, Carlos Ruiz Puig, Aileen Ryan, Benjamin Schuster-Böckler, Paulina Siejka-Zielińska, Chunxiao Song, Marketa Tomkova, Benoit J. Van den Eynde, Gergana Velikova, Ralph R. Venhaus, Michael J. White, Phil F. Xie

**Affiliations:** 1 Ludwig Institute for Cancer Research, University of Oxford, Oxford, UK; 2 Wellcome Centre for Human Genetics, University of Oxford, Oxford, UK; 3 NIHR Oxford Biomedical Research Centre, Oxford University Hospitals NHS Foundation Trust, John Radcliffe Hospital, Oxford, UK; 4 Big Data Institute, University of Oxford, Oxford, UK; 5 Department of Pathology and Molecular Medicine, University of British Columbia, Vancouver, Canada; 6 Department of Computer Science, University of British Columbia, Vancouver, Canada; 7 Department of Molecular Oncology, BC Cancer, Vancouver, Canada; 8 Cancer Research UK Southampton Experimental Cancer Medicine Centre, Cancer Sciences Unit, Faculty of Medicine, University of Southampton, Southampton, UK; 9 Department of Oncology, University of Oxford, Oxford, UK; 10 Early Phase Clinical Trials Unit, Cancer & Haematology Centre, Churchill Hospital, Oxford, UK; 11 Oncology Clinical Trials Office (OCTO), Department of Oncology, University of Oxford, Oxford, UK; 12 Ludwig Cancer Research, New York, NY, USA; 13 Ludwig Institute for Cancer Research, Brussels, Belgium; 14 de Duve Institute, Université Catholique de Louvain, Brussels, Belgium; 15 Cancer Sciences Unit, University of Southampton and Cancer Care Group, University Hospital Southampton NHS Foundation Trust, Southampton, UK

**Keywords:** esophageal cancer, immune priming, immune checkpoint inhibitors, immunochemotherapy, predictive biomarkers, tumor associated monocytes, tumor mutational burden, molecular profiling, single-cell RNA-sequencing atlas, cell type deconvolution

## Abstract

For inoperable esophageal adenocarcinoma (EAC), identifying patients likely to benefit from recently approved immunochemotherapy (ICI+CTX) treatments remains a key challenge. We address this using a uniquely designed window-of-opportunity trial (LUD2015-005), in which 35 inoperable EAC patients received first-line immune checkpoint inhibitors for four weeks (ICI-4W), followed by ICI+CTX. Comprehensive biomarker profiling, including generation of a 65,000-cell single-cell RNA-sequencing atlas of esophageal cancer, as well as multi-timepoint transcriptomic profiling of EAC during ICI-4W, reveals a novel T cell inflammation signature (INCITE) whose upregulation correlates with ICI-induced tumor shrinkage. Deconvolution of pre-treatment gastro-esophageal cancer transcriptomes using our single-cell atlas identifies high tumor monocyte content (TMC) as an unexpected ICI+CTX-specific predictor of greater overall survival (OS) in LUD2015-005 patients and of ICI response in prevalent gastric cancer subtypes from independent cohorts. Tumor mutational burden is an additional independent and additive predictor of LUD2015-005 OS. TMC can improve patient selection for emerging ICI+CTX therapies in gastro-esophageal cancer.

## Highlights

• An EAC immunochemotherapy (ICI+CTX) trial: four weeks of ICI (ICI-4W), then ICI+CTX
• A T/NK cell gene signature (INCITE) is upregulated in patients responding to ICI
• High tumor monocyte content and mutational burden predict ICI+CTX outcomes
• This biomarker combination also shows promise for EBV-/MSS gastric cancer

## Graphical abstract

**Figure:**
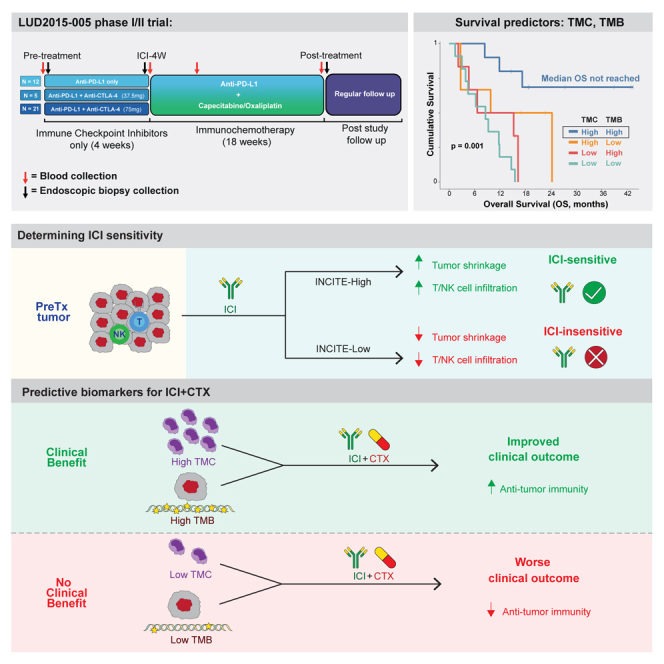

## Teaser

Carroll et al. report that four weeks of immune checkpoint inhibitors is sufficient to induce tumor shrinkage in esophageal adenocarcinoma patients upregulating the “INCITE” gene signature. Integration of single-cell RNA sequencing and bulk RNA sequencing through deconvolution identified tumor monocyte content and tumor mutational burden as independent and complementary predictive biomarkers for immunochemotherapy.

## Introduction

Immunotherapy with immune checkpoint inhibitors (ICIs) has emerged as the fourth pillar of cancer care, alongside chemotherapy (CTX), radiotherapy, and surgery. Increasing numbers of patients have been treated with ICIs, either alone or in combination with concurrent CTX (immunochemotherapy [ICI+CTX]). Despite exciting improvements in patient outcomes with ICIs, only a minority of patients attain long-term benefits with these agents.^1^ With the expanding number of regulatory approvals and thousands of ongoing clinical trials for immunotherapies,^2^ identifying patients who are most likely to benefit from ICI-based therapies remains a key challenge.

Previous studies identify a number of key predictors of response to ICI, including tumor mutational burden (TMB), markers of T cell inflammation, and expression of targeted checkpoint molecules, such as PD-1 or PD-L1.^3^ Biomarkers predicting favorable outcomes on ICI may not predict outcomes with CTX alone or ICI+CTX, as ICI acts by stimulating the immune system, whereas CTX with DNA damaging agents can impair both cancer and non-malignant immune cells. Therefore, uncoupling ICI-specific signals from the confounding effect of CTX is a crucial step in identifying biomarkers that could help select patients for the growing range of ICI+CTX indications. TMB remains one of the most well-studied genomic predictors of ICI response, although this biomarker may not have equal utility in all cancer types.^4^ Disambiguating the role of TMB, and finding novel biomarkers to complement its predictive utility, would enhance our ability to select patients for immunochemotherapy.

Esophageal cancer is the sixth leading cause of cancer mortality.^5^ The predominant subtype in the Western world, esophageal adenocarcinoma (EAC), is among the cancer types with the highest increase in incidence over the past few decades.^6^^,^^7^^,^^8^^,^^9^^,^^10^ About 40% of esophageal cancers present with distant metastases at diagnosis.^11^ For these inoperable patients, median OS with conventional first-line fluoropyrimidine and platinum CTX is less than one year.^12^^,^^13^^,^^14^^,^^15^^,^^16^ In 2021, the U.S. Food and Drug Administration (FDA) approved first-line ICI+CTX regimens using αPD-1 ICI with platinum/fluoropyrimidine CTX for inoperable gastro-esophageal cancers.^17^^,^^18^ However, conventional predictive biomarkers for ICI have shown variable predictive utility for this setting. High PD-L1 expression, assessed histologically using the combined positive score (CPS) method, shows associations with increased survival benefit when adding ICI to CTX in some first-line phase III trials in advanced gastro-esophageal cancers,^19^^,^^20^ and some regions use CPS thresholds to determine αPD-1 ICI+CTX eligibility in these cancers.^21^ However, PD-L1 expression is not associated with improved outcomes in other αPD-1 ICI+CTX gastro-esophageal cancer trials.^22^^,^^23^ In the first-line setting, one study reports an increased magnitude of benefit in TMB-high patients for ICI+CTX compared with CTX^24^; whereas in pre-treated gastro-esophageal cancers, TMB either fails to show a significant association with ICI outcomes^25^^,^^26^ or does not maintain significance following multivariable regression.^27^ Microsatellite instability (MSI) and Epstein-Barr virus (EBV)-associated gastro-esophageal cancers tend to respond particularly well to ICI-containing regimens,^28^^,^^29^ but these features are only present in a fraction of gastric cancers (GC), and are rare or absent in EAC.^30^ These findings highlight the need to identify additional biomarkers that can identify gastro-esophageal cancer patients who would benefit most from ICI+CTX, particularly for the rapidly growing EAC patient population.

The phase I/II LUD2015-005 trial, initiated in 2015, provided a unique opportunity to address these challenges. Patients with inoperable esophageal cancers, predominantly EAC, were treated with ICI alone for a window of four weeks (ICI-4W) prior to ICI+CTX. Paired biopsies of malignant and normal gastrointestinal (GI) tissues were taken before, during, and after treatment. This design enabled comprehensive clinical and molecular profiling throughout treatment using whole genome sequencing (WGS), single-cell RNA sequencing (scRNA-seq), and bulk RNA sequencing (bulk RNA-seq). Analysis of sequential tumor biopsies collected before and after ICI-4W, before the confounding influence of CTX, identified treatment-responsive molecular signatures that correlated with response and resistance to first-line ICI-only in EAC and predicted long-term ICI outcomes in other settings. Integrating scRNA-seq and bulk RNA-seq through deconvolution also uncovered the key role of intratumoral cell type composition in predicting long-term outcomes on ICI-based therapies, both in LUD2015-005 and in a validation cohort of ICI-treated GC. To ensure predictive biomarkers identified in the LUD2015-005 study were specific to ICI-containing regimens, their predictive utility was also tested in EAC patients from The Cancer Genome Atlas (TCGA)^30^ and International Cancer Genome Consortium (ICGC),^31^ whose reported pharmacological treatment consisted largely of conventional CTX. The relationship of cell composition biomarkers with TMB was also investigated, aiming to establish pre-treatment biomarkers that could complement TMB to improve the prediction of long-term outcomes of ICI+CTX in gastro-esophageal cancers.

## Results

### Treating esophageal cancer with ICI-4W prior to ICI+CTX in the LUD2015-005 trial

**Figure 1:**
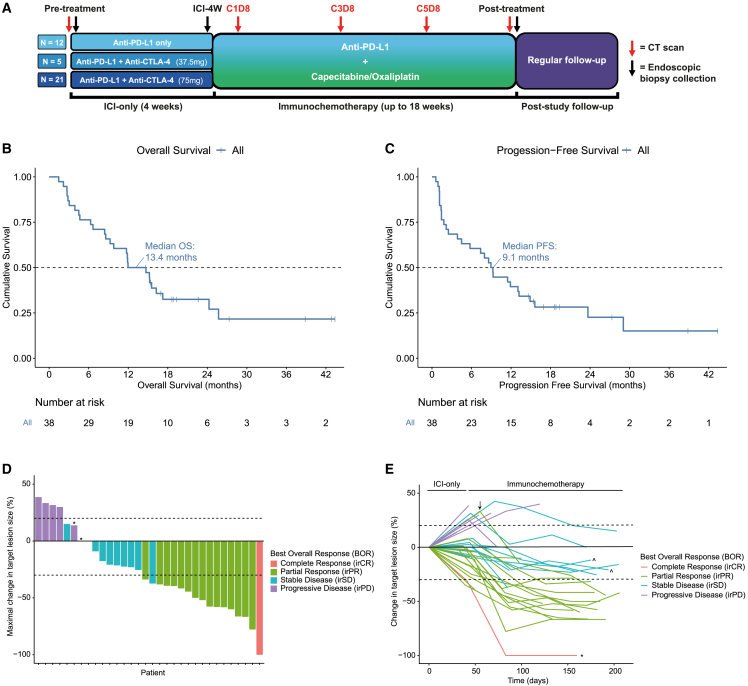
LUD2015-005 design and clinical outcomes (see also Figure S1) (A) LUD2015-005 timeline of treatment, sampling, and CT response-assessment events. On-treatment CT scans are named according to their timing relative to ICI+CTX (C1D8 = cycle 1, day 8). (B and C) Kaplan-Meier curves for (B) OS and (C) PFS for all LUD2015-005 inoperable patients. Risk table (below) shows number of patients with ongoing survival at each timepoint. (D) Maximal target lesion shrinkage (sum of diameters; not including new/non-measurable lesions) attained at any CT scan during the study. Bars are colored by irRECIST BOR. Three patients (1 irCR, 2 SD) not shown due to absence of measurable target lesions. Three patients who passed away from clear clinical progression prior to any on-treatment CT (clinical PD) also not shown. ^∗^Patients with irSD of target lesions but unequivocal progression due to new lesions (overall irRECIST response of irPD). (E) Spider plot showing CT-assessed change in target lesion size from the pre-treatment scan for each patient throughout the study. Certain patients highlight the difficulty in summarizing outcomes using response: ↓ = lesions grew during ICI-4W, but eventually attained irPR during immunochemotherapy; ^∗^ = irCR but average PFS (progressed at 9.3 months); ˆ = PFS>12 months but no irRECIST response (two additional cases had non-assessable target lesion sizes).

The phase I/II LUD2015-005 trial (NCT02735239, EudraCT 2015-005298-19) was designed to understand ICI and ICI+CTX responses in previously untreated inoperable esophageal cancers. Treatment began with a four-week ICI-only window (ICI-4W), followed by six cycles of ICI+CTX. During ICI-4W, either αPD-L1 (n = 12) or αPD-L1 with a single priming dose of αCTLA-4 (n = 26) was administered; αPD-L1 was then given alongside oxaliplatin and capecitabine during the ICI+CTX phase. Endoscopic biopsies and CT scans were collected before and throughout treatment (Figure 1A).

The demographic and clinical characteristics of the 38 patients (35 EAC; 3 esophageal squamous cell carcinoma [ESCC]) in the intention-to-treat population (ITT) for the LUD2015-005 inoperable cohorts are provided in Table S1. Primary outcomes were related to safety (see STAR Methods). All ITT patients reported at least one treatment-emergent adverse event (TEAE), with 29 (76.3%) reporting grade 3 or higher TEAEs (Table S2). No dose-limiting toxicities were encountered during the dose escalation phase.

Secondary outcomes were OS, progression-free survival (PFS), and tumor response measured by irRECIST.^32^ Median OS and PFS in the ITT population were 13.4 and 9.1 months, respectively (Figures 1B and 1C, and Table 1). Comparing the OS of LUD2015-005 patients with that of a propensity-matched cohort of CTX-treated patients from SEER,^33^ a US-based registry of cancer cases, suggested a potential trend toward increased OS in the LUD2015-005 cohort, but this trend did not reach statistical significance, possibly due to the small cohort size of LUD2015-005 (Figure S1A).

**Table 1:** Clinical outcomes of inoperable LUD2015-005 cohorts

ICI Agent	αPD-L1 only		αPD-L1+ 37.5mg αCTLA-4		αPD-L1+ 75mg αCTLA-4		All	
	Full ITT (n = 12)	EAC only (n = 11)	Full ITT (n = 5)	EAC only (n = 5)	Full ITT (n = 21)	EAC only (n = 19)	Full ITT (n = 38)	EAC only (n = 35)
irRECIST BOR								
Response (irCR/irPR)	5 (41.7%)	5 (45.5%)	2 (40%)	2 (40%)	10 (47.6%)	9 (47.4%)	17 (44.7%)	16 (45.7%)
Non-response (irSD/irPD)	7 (58.3%)	6 (54.5%)	3 (60%)	3 (60%)	11 (52.3%)	10 (52.6%)	21 (55.3%)	19 (54.3%)
Clinical benefit (>12 months PFS)								
Yes (CB)	5 (41.7%)	5 (45.5%)	0	0	10 (47.6%)	9 (47.4%)	15 (39.5%)	14 (40%)
No (NCB)	7 (58.3%)	6 (54.5%)	5 (100%)	5 (100%)	10 (47.6%)	9 (47.4%)	22 (57.9%)	20 (57.1%)
Not assessable^a^	0	0	0	0	1 (4.8%)	1 (5.3%)	1 (2.6%)	1 (2.9%)
Overall survival (months)								
Median (95% CI^b^)	13.5 (6.7-NA^c^)	11.8 (6.7-NA^c^)	8.6 (4.5-NA^c^)	8.6 (4.5-NA^c^)	15.6 (11.9-NA^c^)	15.6 (11.9-NA^c^)	13.4 (9.3–24.3)	11.9 (8.6–24.3)
Progression-free survival (months)								
Median (95% CI^b^)	7.5 (2.2-NA^c^)	9.3 (2.5-NA^c^)	7.4 (4.5-NA^c^)	7.4 (4.5-NA^c^)	11.9 (3.9-NA^c^)	11.9 (9-NA^c^)	9.1 (4.5–15.6)	9.3 (5.8–15.6)

Outcome statistics for ICI treatment subgroups and all treated patients (rightmost). Within each population, values for the full ITT population (n = 38) and the 35 adenocarcinomas are shown.

a See STAR Methods.

b CI: Confidence Interval.

c NA: CI upper bound not reached due to ongoing patient survival. See also Table S8.

In the ITT population, 17/38 patients (44.7%) showed an irRECIST response, defined as a 30% reduction in tumor burden from pre-treatment measurements^32^ (Figure 1D and Table 1). Many patients already displayed tumor shrinkage during ICI-4W; indeed, three met the definition for response immediately following the ICI-4W window, suggesting four weeks of ICI-only is sufficient to induce tumor shrinkage in a subset of patients (Figure S1B). Interestingly, some patients responded differently in the different phases of therapy. A few patients with unconfirmed progression (≥20% increase in tumor burden) during ICI-4W attained stable disease or responses during ICI+CTX (Figure 1E), either representing ICI progression followed by subsequent CTX response, or instances of pseudoprogression, where ICI-induced immune influx preceding an eventual anti-tumor response causes a transiently increased tumor size.^34^

Four irRECIST non-responders nonetheless experienced prolonged PFS (>12 months) (Figure 1E). As the strength of association between response and long-term survival may vary, an additional “clinical benefit” (CB) outcome metric was established to denote long-term disease control on this treatment protocol, following previous ICI biomarker studies.^35^^,^^36^ Patients attaining 12 months of PFS were deemed to have attained CB, while the rest were classified as no clinical benefit (NCB). 15 of 37 (40.5%) assessable LUD2015-005 patients attained CB (Table 1). Due to its definition, CB classification was more closely linked with long-term survival outcomes than irRECIST response (Figures S1C and S1D). In summary, four weeks of first-line ICI-only was sufficient to induce tumor shrinkage in some inoperable esophageal cancer patients, while around 40% achieved CB on subsequent αPD-L1 ICI+CTX.

### ICI-4W treatment induces INCITE signature, an on-treatment marker of ICI efficacy

**Figure 2:**
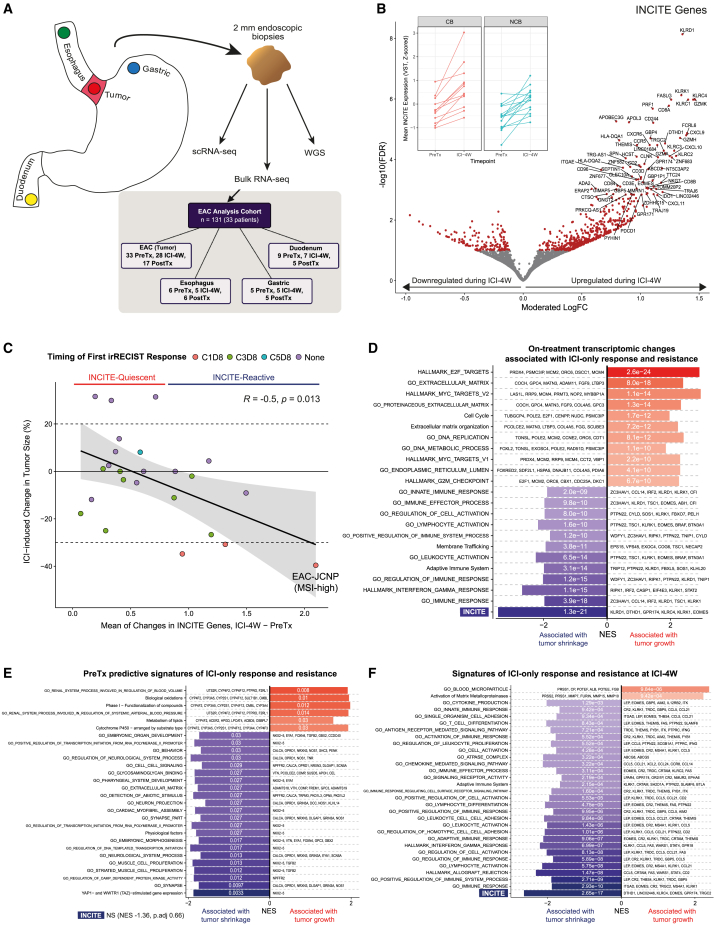
Molecular features of response and resistance to ICI (see also Figures S2 and S3) (A) Top: Endoscopic sampling sites for biomarker analyses. Esophageal and gastric biopsies were taken ≥2cm away from tumor and gastro-esophageal junction. Bottom: Summary of bulk RNA-sequencing dataset. (B) Differential expression between PreTx and ICI-4W for patients with biopsies at both timepoints (n = 28), controlling for patient-specific effects (see STAR Methods). Moderated log fold change and FDR are shown; the top 70 significant genes are labeled. All significant DEGs (FDR<0.1) are in red. Inset: Mean variance stabilization transformed (VST) expression of INCITE genes (top 70 DEGs), *Z* score normalized and displayed with CB and NCB facets. Lines connect values for the same patient across timepoints. (C) Mean VST expression change of INCITE genes (scaled, without centering) compared to percentage change of tumor size (target lesions) at the C1D8 scan from PreTx. Points are colored by the timing of first report of irRECIST response, and the MSI tumor is labeled. Pearson correlation statistics are displayed. (D–F) Fast gene set enrichment analysis (FGSEA) results showing the most significantly enriched pathways. Bar length represents normalized enrichment score (NES); color reflects the adjusted p value (labeled). For (D), the FGSEA test statistic was the correlation coefficient between changes in VST-normalized gene expression (ICI-4W–PreTx) and changes in tumor size during ICI-4W. For (E) (PreTx) and (F) (ICI-4W), the test statistic was moderated log fold change calculated by DESeq2 at each timepoint using scaled ICI-4W tumor size changes (continuous variable) in the design formula.

Molecular analyses were limited to EAC patients (35 of 38 in this cohort) with available biopsies. The LUD2015-005 trial design and sample collection protocol provided a unique opportunity to elucidate early response and resistance mechanisms for first-line ICI-only in EAC. We generated a multi-timepoint bulk RNA-seq dataset using tumor samples from 33 EAC patients and paired normal GI tissues from a subset of patients (Figure 2A). Paired tumor transcriptomes from both before (PreTx) and after (ICI-4W) the initial ICI-4W window were available for 28 EAC patients. Assessing transcriptional changes between PreTx and ICI-4W, we found that the top differentially expressed genes (DEGs) upregulated during ICI-4W were dominated by markers of cytotoxic inflammation, including T cell chemokines (*CXCL9*, *CXCL10*, and *CXCL11*), T/NK cell markers (*CD2*, *CD3D/E*, *CD8A/B*, killer cell lectin-like receptor [KLR] family, *NKG7*, and*TRGC2*), cytotoxic effector molecules (*GZMA*, *GZMH*, *GZMK*, *PRF1*, and *FASLG*), and markers of CD8^+^ tissue-resident memory T cells (*ITGAE*/CD103 and *ZNF683*/Hobit) (Figure 2B and Table S3). As the top 70 DEGs were dominated by these upregulated cytotoxic markers, we used these genes to define an ICI-responsive gene signature which we termed INCITE (ImmuNe Checkpoint Inhibitor-induced T/NK-cell Enrichment).

Many INCITE genes were significantly upregulated by both αPD-L1 and αPD-L1+αCTLA-4 treatment during ICI-4W (Figure S2A). Indeed, upregulation of INCITE genes was observed in nearly all LUD2015-005 patients at ICI-4W, although the extent varied considerably (Figures 2B and 2C). The extent of INCITE gene upregulation was significantly correlated with shrinkage in tumor burden during the ICI-4W window, suggesting the induction of this transcriptional signature was linked to overall ICI efficacy. Ordering patients by their mean upregulation of INCITE genes, we subdivided patients into INCITE-reactive (greater ICI-induced INCITE upregulation), and INCITE-quiescent (less upregulation) groups. All three patients with unconfirmed irRECIST progression during ICI-4W were INCITE-quiescent, suggesting these cases did not represent pseudoprogression due to intratumoral immune cell influx, while the INCITE-reactive group included all three patients exhibiting irRECIST responses following ICI-4W (Figures 2C and S2B). The patient with the greatest INCITE upregulation (EAC-JCNP) also showed the most tumor shrinkage during ICI-4W. These findings suggest intratumoral INCITE upregulation is a strong correlate of early responses to ICI-only.

Gene set enrichment analysis (GSEA) showed that upregulation of various inflammatory and interferon gamma-related signatures was strongly linked with tumor shrinkage (Figures 2D, S2C, and S2D). INCITE upregulation outperformed other inflammatory gene sets as a correlate for ICI-4W response (FDR = 1.3e-21; Figure 2D). Conversely, upregulation of E2F targets (e.g., *PRDX4*, *MCM2*; FDR = 2.6e-24) and extracellular matrix genes (e.g., *COCH*, *MATN3*; FDR = 8.0e-18) were strongly associated with tumor growth and resistance to ICI-4W (Figures 2D, S2E, and S2F). These results show that four weeks of ICI is sufficient to induce appreciable T cell inflammation in this setting, best captured by the INCITE signature, and that on-treatment INCITE upregulation is a key marker of early anti-tumor immune responses during ICI-only.

As a smaller gene set would be more amenable to potential clinical applications, we tested the utility of all INCITE subsets and found that upregulation of the top 12 INCITE genes (INCITE-12) maintained a strong correlation with tumor shrinkage while preserving robustness against randomly generated noise (Figures S2G and S2H, and Table S3). Comparing INCITE-12 against five other inflammatory signatures used as predictive biomarkers in clinical ICI research^37^^,^^38^^,^^39^^,^^40^ revealed that only INCITE-12 upregulation was significantly correlated with ICI-induced tumor shrinkage in LUD2015-005 EAC patients (Table S3).

While INCITE-12 upregulation was greater in early responders to ICI-only in LUD2015-005, it was not associated with long-term CB following ICI+CTX (Figure S2I), perhaps due to some ICI-resisting tumors responding to the CTX component of therapy. Therefore, to further assess the utility of INCITE-12 as an ICI-only biomarker, we assessed INCITE-12 upregulation alongside the five other inflammatory signatures in an ICI-naïve melanoma cohort receiving αPD-1 ICI, where sampling was conducted before and after a similar four-week ICI-only window.^41^ In this cohort, INCITE-12 upregulation during the first four weeks was significantly associated with overall ICI response and benefit (Figure S2J), showing the strongest association with these metrics of any gene set tested (Table S3). In another cohort of patients from the same report who had previously progressed on αCTLA-4 ICI, no signature showed a significant association with outcomes, suggesting INCITE-12 was most suitable for the ICI-naïve setting (Table S3). Early upregulation of INCITE-12 is a promising on-treatment biomarker for overall ICI-only outcomes.

### Immune responses associated with early ICI outcomes are detectable with single-timepoint ICI-4W sampling

We next examined whether PreTx expression of INCITE or other genes could predict ICI-4W outcomes. DESeq2 was used to identify PreTx genes significantly associated with changes in tumor burden during ICI-4W as a continuous variable. DEGs and GSEA revealed that neither T cell inflammation signatures such as INCITE (FDR = 0.66) nor PreTx PD-L1 expression (FDR = 0.93) predicted tumor shrinkage following ICI-4W (Table S3). Instead, PreTx expression of neural and muscle development genes was associated with ICI-4W tumor shrinkage, and CYP450 genes, a superfamily involved in xenobiotic metabolism, with tumor growth (Figures 2E and S3A–S3C). Pathways in PreTx GSEA had weaker associations with ICI-4W outcomes than the dynamic analysis, suggesting the strength of association between PreTx expression and ICI-4W outcomes may be limited. Commonly assessed transcriptional biomarkers, including PD-L1 and T cell inflammation signatures, may not be strongly predictive of first-line αPD-L1 ICI outcomes in inoperable EAC.

In contrast, in single-timepoint analyses of ICI-4W biopsies, many genes showed highly significant associations with ICI-induced changes in tumor burden. GSEA showed ICI-4W expression of several inflammatory signatures was strongly linked with tumor shrinkage, recapitulating the dynamic analysis; of these, INCITE again showed the most significant association. γδ T cell markers (*TRDC*, *TRGC1*, and *TRGC2*) appeared to be key contributors to the enrichment of inflammatory gene signatures in this analysis (Figures 2F and S3E). Higher ICI-4W expression of extracellular matrix (ECM) genes, including trypsinogens (*PRSS1* and *PRSS2*) and matrix metalloproteases, and genes encoding cancer antigens (*CTAG2*, *SAGE1*, *MAGEA1*/*A4/A10*, *POTEE*, and *PRAME*) was significantly associated with ICI resistance and tumor growth during ICI-4W (Figures 2F and S3D–S3F, and Table S3). Preserved expression of these immunogenic cancer-specific antigens at ICI-4W likely represents insufficient generation of anti-tumoral immune responses in ICI-resistant patients. Together, single-timepoint sampling after ICI-4W enables the evaluation of treatment-emergent signatures of ICI response (INCITE) and resistance (ECM and cancer antigens) that are comparatively absent before treatment. ICI-4W sampling could supplement radiological response to help predict long-term ICI-only outcomes soon after treatment onset.

### IPRES signatures at ICI-4W mark INCITE-quiescent tumors resisting ICI treatment

As extracellular matrix genes were associated with ICI resistance in dynamic and ICI-4W analyses, we assessed innate PD-1 resistance (IPRES) signatures, a group of gene pathways including stromal modules reported to predict ICI resistance in melanoma.^42^ INCITE-quiescent patients showed higher ICI-4W IPRES levels compared to INCITE-reactive patients (Figure S3G). This finding was not only found for the stromal IPRES modules: a similar trend could also be seen for hypoxia, EMT/metastasis, and TGFβ IPRES modules (Figure S3H). Unlike the original melanoma study, in this EAC cohort, only ICI-4W IPRES scores were associated with ICI resistance; PreTx IPRES scores showed no significant association (Figure S3G).

A subset of INCITE-quiescent patients with high ICI-4W IPRES levels attained CB during the ICI+CTX phase (Figure S3I). This could signify that ICI+CTX can overcome ICI-only resistance in some patients, but could also be due to delayed ICI-only responses. As our study did not include an ICI-only arm, future studies are needed to fully interpret this finding. Regardless, these findings suggest that while IPRES and INCITE signatures mark early response and resistance to ICI-only in EAC, they are not sufficient to explain long-term ICI+CTX outcomes.

### Innate immune signatures predict clinical benefit on immunochemotherapy

**Figure 3:**
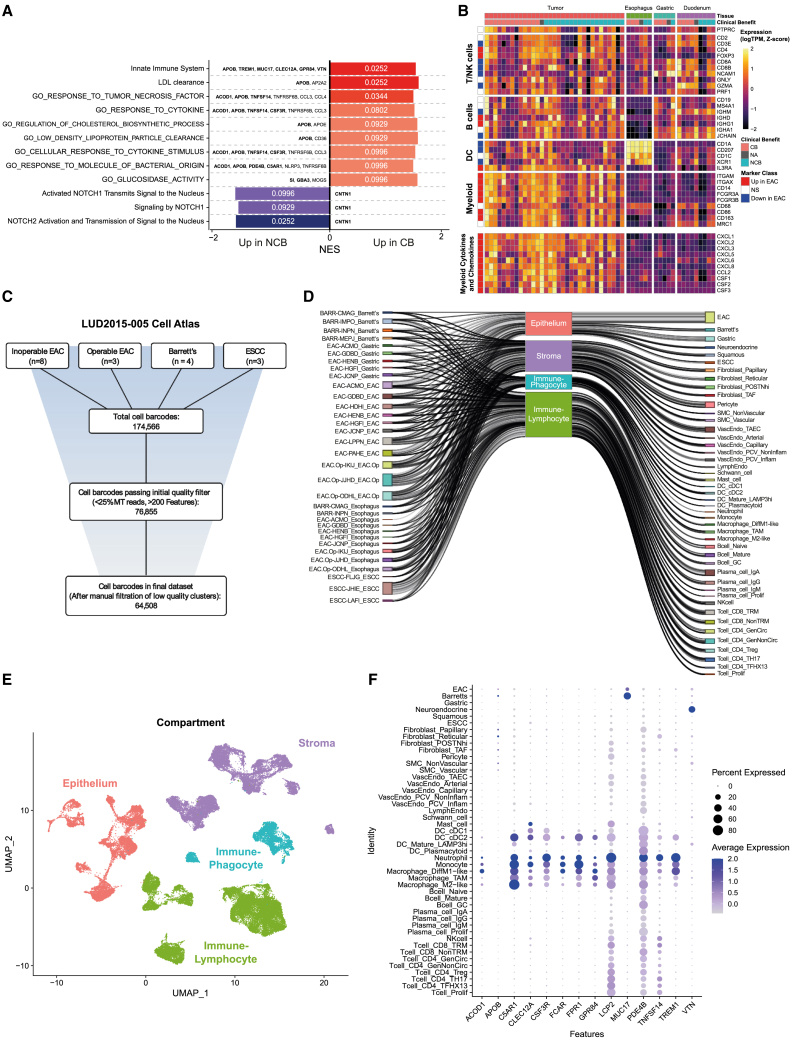
Myeloid phenotype in EAC predicts CB on ICI+CTX (see also Figures S4 and S5) (A) FGSEA results showing significantly enriched PreTx pathways in CB and NCB (FDR<0.1). Bar length represents NES; color reflects adjusted p value (labeled). Test statistic is DESeq2 moderated log fold changes (CB vs. NCB). (B) Heatmap of Z-score-normalized logTPM expression for markers of general immune infiltration (*PTPRC*/CD45), T/NK cells, B cells, dendritic cells (DC), other myeloid cells, and a panel of myeloid-targeted cytokines and chemokines, across different PreTx tissues. Genes significantly (FDR<0.1) up or down in EAC compared to other tissues are labeled. (C) Preprocessing summary for the LUD2015-005 atlas showing cell barcodes remaining after each filtration step (see STAR Methods). Total cell barcodes are the true cell barcodes called by Cell Ranger (filtered feature-barcode matrix). (D) Sankey plot illustrating the contribution of each patient-tissue type combination to the four broad cellular compartments and their constituent cell types. (E) Uniform manifold approximation and projection (UMAP) dimensionality reduction of all cells surviving quality control (QC) and filtering in the LUD2015-005 atlas, colored by cellular compartments. Batch effects due to dissociation method were first removed using FastMNN. (F) Dot plot of significant PreTx CB-associated DEGs in innate immune-related gene sets (from Figure 3A). For each gene, dot color represents average expression in each cell type (scaled and log-normalized), while size reflects the percentage of cells with detectable expression in each cell type.

Given its clinical use in αPD-1 ICI+CTX, the association of PD-L1 expression with ICI+CTX outcomes was first assessed, but no significant association with survival in LUD2015-005 was found (Table S3). We, therefore, conducted differential expression between CB and NCB PreTx tumors to identify biomarkers predictive of ICI+CTX outcomes. Surprisingly, DEGs and gene sets significantly associated with CB were related to the innate immune system, particularly myeloid markers (*TREM1*, *ACOD1*, *TNFSF14*, and *CSF3R*), rather than T cell inflammation markers and checkpoint molecules more commonly described as predictive of positive outcomes in ICI-based regimens (Figures 3A and S4A, and Table S3).^3^^,^^43^^,^^44^ To understand whether the importance of innate immune signatures might be due to specific features of the EAC microenvironment, we compared PreTx expression of immune markers between EAC (n = 33) and paired normal GI tissue biopsies (esophagus, gastric, and duodenum) from a subset of patients (n = 5–9; Figure 2A) and observed a significantly higher expression of myeloid markers (particularly monocyte, macrophage, and neutrophils: *CD14*, *FCGR3A/*CD16A, *CD163*, and *FCGR3B/*CD16B) in EAC (Figure 3B). Contrastingly, dendritic cell, T cell, and B cell markers were expressed similarly to normal GI tissue. Cytokines and chemokines known to promote infiltration of myeloid cells or modulate their function (*CXCL3*, *CCL2*, and *CSF1*/M-CSF) were also highly enriched in EAC, and increased expression of these genes was strongly correlated with increased myeloid cell markers (Figures 3B and S4B). Transcriptomes from TCGA and Genotype-Tissue Expression (GTEX) databases also showed significant enrichment for myeloid markers and cytokines and chemokines in EAC compared with normal GI tissues (Figure S4C). Together, increased myeloid infiltrate is a defining feature of the EAC microenvironment consistent with the enhanced expression of relevant cytokines and chemokines, and it appears a skew in composition or phenotype of this characteristic myeloid enrichment plays an important role in determining ICI+CTX outcomes.

### Generating a 65,000-cell upper GI cell atlas and establishment of a deconvolution workflow

To identify which myeloid cell types may influence ICI+CTX outcomes, we integrated evidence from scRNA-seq and bulk RNA-seq. We first generated the LUD2015-005 upper GI cell atlas, a 65,000-cell scRNA-seq dataset derived from diseased and normal gastro-esophageal tissues collected from 18 patients, including inoperable and operable EAC, ESCC, and the pre-malignant lesion Barrett’s esophagus (Figures 3C–3E). We identified 46 major cell types, with good representation of epithelial, stromal, and lymphocytic and phagocytic immune cell compartments (Figures S4D–S4G and S5). LUD2015-005 atlas data verified INCITE genes were specifically expressed in T cells and NK cells (Figure S4H) and confirmed increased levels of monocytes, macrophages, and neutrophils in EAC (Figure S4I). Many cell types from EAC samples expressed myeloid-targeted cytokines and chemokines in this dataset: particularly myeloid cells themselves, but also a subset of tumor cells (Figure S4J). Finally, this atlas showed that the innate immunity DEGs associated with CB in bulk RNA-seq differential expression were mainly expressed by monocytes, neutrophils, and M1-like macrophages (Figure 3F), suggesting that the skew of the characteristic myeloid infiltrate in EAC toward one or more of these cell types predicted better ICI+CTX outcomes.

To further define which myeloid cell type was primarily responsible for the association with CB, we conducted deconvolution to compute the cellular composition of trial-derived bulk RNA-seq samples. To select the deconvolution algorithm with the best performance using the LUD2015-005 scRNA-seq atlas as reference, we first pooled single-cell transcriptomes to create 80 pseudobulk RNA-seq samples with known cellular composition. We compared deconvolution estimates with the ground-truth pseudobulk composition and found that BayesPrism^45^ had the smallest median error and highest median correlation of all algorithms tested (see STAR Methods; Figure S6 and Table S4). We, therefore, selected BayesPrism for deconvolution using the LUD2015-005 atlas. In a previously published RNA-seq dataset,^46^ this deconvolution approach was able to differentiate the epithelial and microenvironment composition of EAC from that of other esophageal tissue types, including the closely related Barrett’s esophagus (Figures S7A–S7C). This BayesPrism deconvolution workflow was therefore selected to estimate the cellular composition of LUD2015-005 bulk transcriptomes.

### TMC identifies gastro-esophageal cancer patients likely to benefit from ICI-based therapy

**Figure 4:**
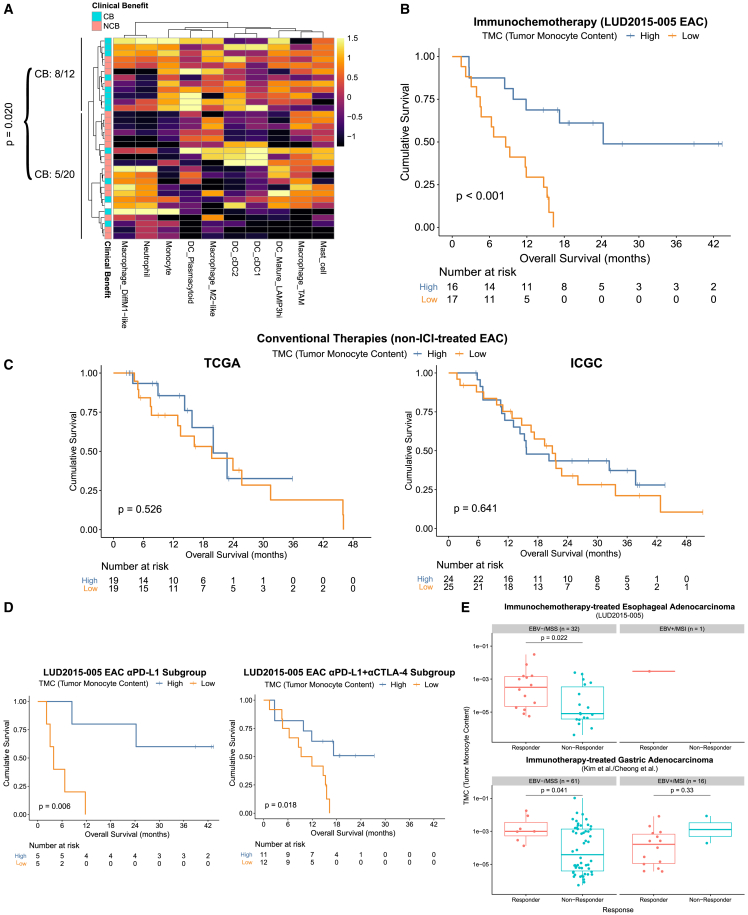
Increased TMC is an ICI+CTX-specific predictor of improved outcomes (see also Figures S7 and S8) (A) Deconvolution-assessed levels of phagocytic immune cell types in LUD2015-005 PreTx EAC biopsies were log10-transformed and scaled by column prior to hierarchical clustering (ward.D linkage). Results are shown as a clustered heatmap, with cells colored according to the scaled deconvolution estimates, and row annotation according to clinical outcomes. CB rates are shown for the monocyte-high cluster and other samples as indicated by the vertical lines. p value (for difference between these rates) by two-proportions z-test. (B) Kaplan-Meier plots showing OS of TMC-high and -low groups from LUD2015-005 EAC patients, as split by the cohort median. p value by log rank test. (C) TMC Kaplan-Meier plots as in (B), but for TCGA (left) and ICGC (right) stage III and IV EACs, used as reference cohorts for non-ICI management. (D) Kaplan-Meier plots comparing TMC-high and TMC-low patients for αPD-L1 (left) and αPD-L1+αCTLA-4 (right) LUD2015-005 treatment subgroups, both split by subgroup median TMC. p values by log rank test. (E) TMC assessment in an independent pooled cohort of ICI-treated GC.^28^^,^^47^ Deconvolution-assessed TMC from LUD2015–005 and GC cohorts are both shown in EBV-/MSS and EBV+/MSI facets, grouped by response. LUD2015-005 response criteria (irRECIST) differed from the GC cohort (RECIST/RECIST v1.1); however binary response calls were the same for irRECIST and RECIST v1.1 criteria in all LUD2015-005 patients shown. p values by Mann-Whitney U-test.

Deconvolution cell composition estimates were conducted on all LUD2015-005 EAC biopsies. At PreTx, assessing the phagocytic immune compartment, a cluster of EACs with high tumor monocyte content (TMC) showed a higher CB rate (8/12 [67%]) than others (5/20 [25%]; Figure 4A). No significant association with outcomes was seen with cell types from epithelial, stromal, or lymphocytic immune compartments (Table S5). Cox regressions on the PreTx level of each cell type revealed that TMC was strongly associated with improved OS, which remained significant following correction for multiple testing (HR: 0.38 (95% CI: 0.22–0.67), p = 0.0008, FDR [Benjamini-Hochberg] = 0.037; Table S5). Other cell types, including neutrophils, M1-like macrophages, and Tregs, showed associations trending toward improved OS but not reaching significance. Using the cohort median to define TMC-high or -low groups effectively stratified patient outcomes (TMC-high median OS: 24.3 months, TMC-low median OS: 8.6 months; Figure 4B). To verify that this predictive biomarker accurately reflected intratumoral monocyte RNA content and not technical artifacts from deconvolution, RNA from peripheral blood monocytes isolated by fluorescence-activated cell sorting (FACS, Figure S7D) was spiked into RNA extracted from esophageal cancer biopsies in known quantities (0–8% of total RNA), and RNA-seq libraries were then prepared. Increasing spiked-in monocyte RNA content was indeed significantly associated with increasing deconvolution-assessed TMC (Figures S7E and S7F).

To assess whether TMC was ICI-specific or a general prognostic biomarker, we analyzed advanced EACs from TCGA and ICGC, where mainly platinum/fluoropyrimidine treatment was reported with no ICI (Table S6). Applying the same deconvolution workflow, we found no significant link between TMC and OS in TCGA or ICGC (Figure 4C), suggesting the association between TMC and survival was specific to ICI-containing regimens including LUD2015-005. Within the LUD2015-005 study, TMC was significantly associated with OS in both αPD-L1- and αPD-L1+αCTLA-4 treatment subgroups, showing this finding was not restricted to either ICI strategy (Figure 4D). Deconvolution-assessed TMC shows powerful potential utility to identify EAC patients most likely to benefit from the addition of ICI to CTX in first-line therapy.

While EBV and MSI are associated with improved outcomes on ICI-based therapies in GC, there are fewer useful predictive biomarkers for the EBV-/microsatellite stable (MSS) subtypes, which comprise the majority of GC. Given the molecular similarity between EAC and the most prevalent EBV-/MSS GC subtype,^30^ we assessed whether TMC could also predict outcomes in an independent pooled cohort of 77 ICI-treated GCs with available RNA-seq and MSI/EBV subtyping.^28^^,^^47^ In EBV-/MSS GC, PreTx TMC was indeed significantly higher in ICI responders than non-responders (p = 0.041), showing similar predictive utility for response as in EBV-/MSS EACs from LUD2015-005 (p = 0.022; Figures 4E and S8A). TMC was not associated with response in EBV+/MSI gastro-esophageal cancers. TMC, therefore, predicts improved outcomes on ICI-containing regimens across independent gastro-esophageal cancer cohorts and could be particularly useful for EBV-/MSS gastro-esophageal cancers.

### TMB and TMC are independent but complementary predictors of immunochemotherapy outcomes

**Figure 5:**
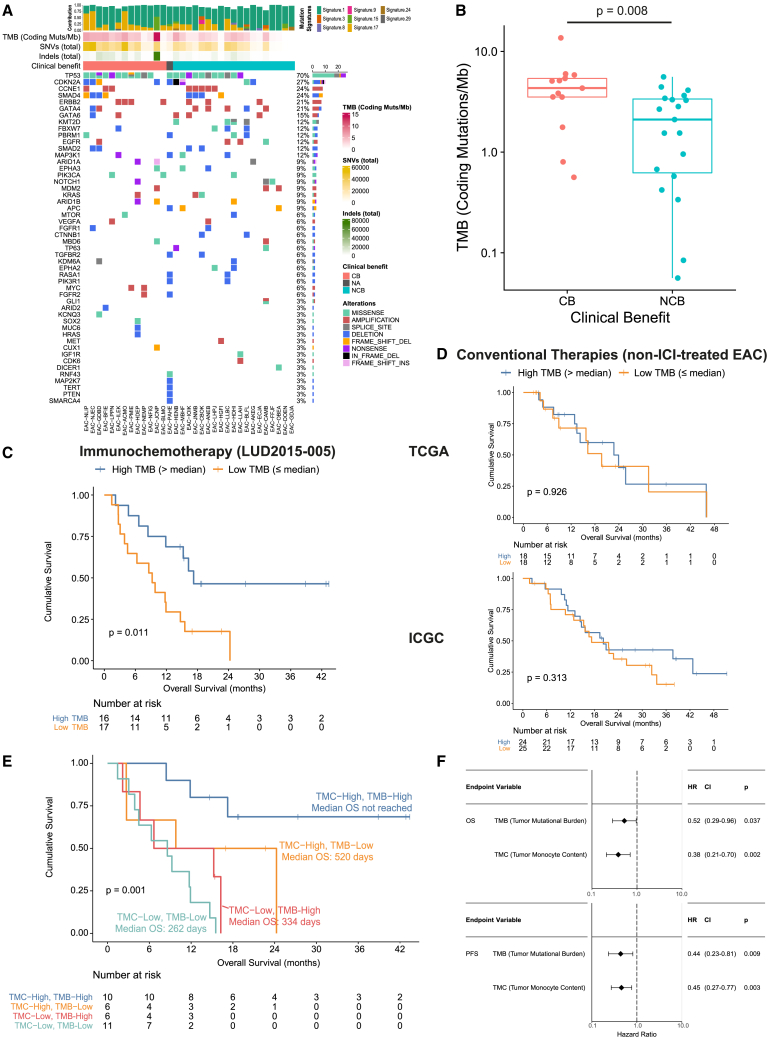
Pre-treatment TMB and TMC are complementary predictive biomarkers for ICI+CTX (see also Figure S8) (A) OncoPrint showing genomic alterations of cancer driver genes in PreTx EAC biopsies. Top barplot indicates the fraction of mutations assigned to predefined single base substitution (SBS) mutational signatures. TMB (coding mutations/Mb) and total SNV and indel numbers are shown. Eight patients had WGS available from multiple PreTx biopsies; for these, TMB, SNVs, and indels represent the average across biopsies, while OncoPrint and SBS signatures were calculated using the union of calls. (B) PreTx TMB for each EAC patient grouped by CB status. p value between groups was calculated by Mann-Whitney U-test. (C and D) Kaplan-Meier plots of TMB-high and TMB-low groups, defined using the cohort median, for (C) LUD2015-005 and (D) non-ICI-treated reference cohorts (as in Figure 4C), split into TCGA (top) and ICGC (bottom). For TCGA and ICGC, only stage III and IV EAC tumors were assessed. p values by log rank test. (E) Kaplan-Meier plots of four subgroups defined by splitting PreTx TMC and TMB values by their respective cohort medians. p value by log rank test (overall difference between the four groups). (F) Forest plot of multivariable Cox regression for OS (top) and PFS (bottom) with PreTx TMC (log10-transformed) and TMB. Both values scaled and centered before regression. Hazard ratio (HR) and 95% CI are shown (HR < 1: association with longer survival; >1: with reduced survival).

TMB is associated with ICI response in several cancers.^3^ We, therefore, conducted whole genome sequencing (WGS) from LUD2015-005 PreTx EAC biopsies (n = 33) to assess TMB and other genomic alterations. The most frequently altered genes in EACs from this cohort were *TP53* (70%) and *CDKN2A* (27%) (Figure 5A and Table S7), similar to previous reports.^31^^,^^48^ Patients attaining CB had significantly higher TMB than NCB patients (median TMB: 4.3 vs. 2.1 non-synonymous coding mutations/Mb [muts/Mb], Figure 5B). Of the 33 EACs assessed, one (from EAC-JCNP) was identified as having MSI; this tumor had the highest TMB (13.7 muts/Mb) in the cohort (see STAR Methods and Table S8). EAC-JCNP attained CB and showed the greatest extent of tumor shrinkage and INCITE upregulation during ICI-4W (Figure 2C).

Stratifying patients by the cohort median TMB (3.3 muts/Mb) showed that TMB-high patients had significantly improved OS (Figure 5C). Cox regression with TMB values also showed a significant association with OS (hazard ratio [95% CI]: 0.50 [0.28–0.90], p = 0.021), which was maintained after excluding the MSI patient (hazard ratio [95% CI]: 0.62 [0.40–0.95], p = 0.029). Like TMC, analysis of advanced EACs from TCGA and ICGC did not show any predictive power for TMB (Figure 5D), suggesting TMB was also more specific to ICI+CTX than CTX alone. Importantly, TMB and TMC were each significantly correlated with ICI-induced tumor shrinkage during the ICI-4W window in LUD2015-005, and their combination showed an even stronger association (Figures S8B–S8D), supporting the specificity of TMB and TMC for ICI-containing protocols. There was no significant correlation between PreTx TMB and TMC in this cohort (Figure S8E), and EAC patients having both TMB and TMC values above their respective cohort medians exhibited greater OS than all other subgroups, suggesting additive predictive power (Figure 5E). In a multivariable Cox regression, both TMB and TMC were significantly associated with longer OS and PFS (Figure 5F), illustrating that these biomarkers are independent predictors of ICI+CTX outcomes and that their combination achieves the best predictive power.

**Figure 6:**
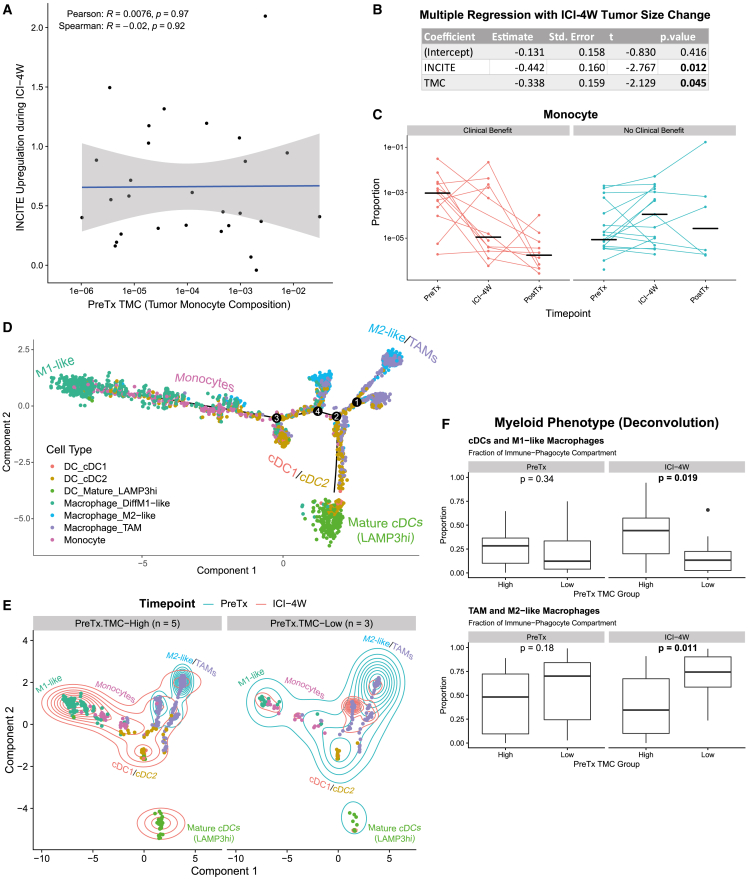
Exploring correlates and potential mechanisms of TMC (see also Table S5) (A) Comparison of INCITE upregulation during ICI-4W and PreTx TMC (log10-transformed). Pearson and Spearman correlation statistics are displayed. (B) Multiple linear regression between PreTx TMC (log10-transformed) and INCITE upregulation with tumor size changes during ICI-4W as dependent variable. Coefficients with p < 0.05 are bolded, representing significant associations with ICI-4W tumor size change. (C) TMC values (log10-transformed) across timepoints, grouped into CB and NCB facets. Each line connects values for the same patient. Crossbars represent median value for that timepoint. (D) Trajectory analysis of non-mast cell phagocytes using Monocle2 (DDRTree). Numbers represent trajectory branch points. Approximate locations of cell types are labeled. (E) Trajectory coordinates from (D) plotted separately for PreTx TMC-high (left, n = 5) and TMC-low tumors (right, n = 3), classified using overall cohort PreTx median. Within each group, the two-dimensional kernel density of cells along the trajectory is shown at PreTx (blue) and ICI-4W (red). Regions enveloped by more contours have more cells present at the specified timepoint. (F) Top: Fraction of immune-phagocyte compartment composed of M1-like macrophages, cDC1, cDC2, and LAMP3-high DCs (phenotypes enriched at ICI-4W in TMC-high tumors in E), assessed by deconvolution. Bottom: as above, but for the fraction of TAMs and M2-like macrophages in immune-phagocyte compartment. p values by Mann-Whitney U-test.

While TMC and T/NK influx (INCITE) both predicted tumor shrinkage during ICI-4W (Figures 2C and S8B), these variables showed no significant correlation, and regression analysis indicated both independently contributed to ICI-induced tumor shrinkage (Figures 6A and 6B). Interestingly, TMC strongly declined during ICI-4W in many patients attaining CB (Figure 6C), indicating that persistence of monocytes throughout treatment was not required to attain prolonged disease control on ICI+CTX. Given this finding and the knowledge that monocytes are able to differentiate into mature macrophage and monocyte-derived conventional dendritic cell (cDC) populations,^49^ we hypothesized that intratumoral monocytes could be stimulated by ICI to differentiate into mature inflammatory myeloid effectors that enhance immune responses to ICI+CTX. Trajectory analysis of myeloid cells from LUD2015-005 scRNA-seq data did suggest that monocytes could transition into several macrophage and dendritic cell populations, with particular proximity to cDCs (especially cDC2), differentiating M1-like macrophages, and other tumor-associated macrophage (TAM) subsets (Figure 6D).

We, therefore, assessed whether ICI generated differing myeloid phenotypes between TMC-high and TMC-low patients. In scRNA-seq data, PreTx intratumoral myeloid cells in both TMC-high and TMC-low subgroups consisted primarily of a general TAM phenotype (Figure 6E). After ICI-4W, most myeloid cells from TMC-low tumors still exhibited TAM and M2-like macrophage phenotypes; contrastingly, in TMC-high patients, ICI strongly shifted the phenotype of myeloid cells toward M1-like macrophage, cDC1, cDC2, and LAMP3-high mature cDC phenotypes at ICI-4W. To confirm in the full cohort, we examined deconvolution results, which showed significantly higher levels of inflammatory myeloid effectors (cDCs and M1-like macrophages) and lower levels of TAM/M2-like macrophages in TMC-high patients than TMC-low at ICI-4W, while the same comparison at PreTx showed no significant differences (Figure 6F and Table S5). These data show that PreTx TMC-high patients generate a significantly more pro-inflammatory myeloid phenotype following ICI-4W.

### Single-cell transcriptomic analysis reveals cell type-specific expression patterns associated with CB

**Figure 7:**
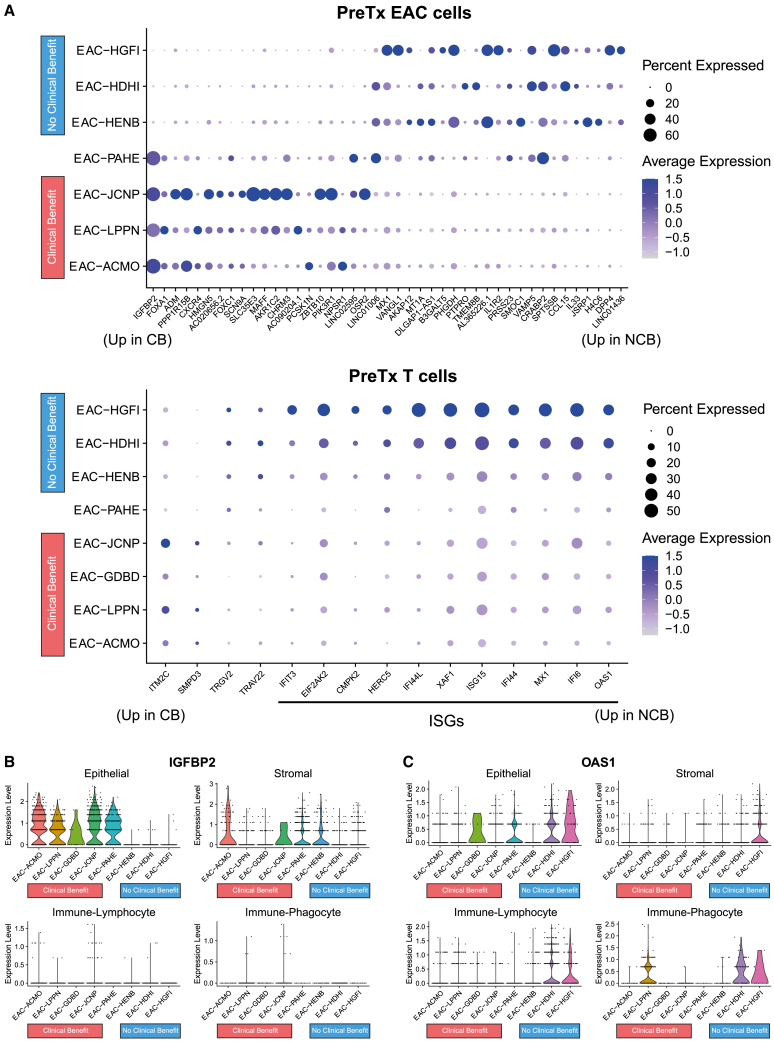
scRNA-seq reveals EAC- and T cell-specific expression patterns predictive of ICI+CTX outcomes (A) Top: EAC-specific PreTx DEGs from pseudobulk differential expression (see STAR Methods) with FDR < 0.1. Dot size represents percentage of cells with any expression; color represents average expression (scaled log-normalized counts). EAC-GDBD was excluded due to insufficient EAC cells. DEGs are sorted by FDR and sign of change (CB leftwards, NCB rightwards). Bottom: as above for T cell-specific DEGs (including EAC-GDBD, having sufficient T cells). Black line: Interferon-stimulated genes (ISGs). (B and C) Violin plot of PreTx expression (log-normalized) of (B) IGFBP2 and (C) OAS1 in single cells from each patient. Plots are faceted by cellular compartments.

We harnessed the detailed information present in scRNA-seq of PreTx EAC biopsies from eight inoperable LUD2015-005 patients (4 CB, 3 NCB, one excluded from CB determination) to investigate whether any gene expression patterns specific to T cells and EAC cancer cells were associated with outcomes. A pseudobulk differential expression approach was employed to prioritize cell type-specific DEGs shared between multiple patients. Analysis of PreTx cancer cell-specific gene expression identified 43 significant DEGs (FDR<0.1). The most significant DEG associated with CB was *IGFBP2*, which was highly expressed in EAC cells from all CB patients, but rarely detectable in EAC cells from NCB patients (Figure 7A). The patient-specific expression pattern for *IGFBP2* was markedly different in epithelium than in stroma, demonstrating the power of scRNA-seq to identify cell type-specific DEGs that otherwise could be masked in bulk transcriptomic approaches (Figure 7B).

Analysis of T cells from PreTx EAC found 15 DEGs (FDR<0.1) between CB and NCB patients, most of which were associated with NCB. NCB-associated T cell-specific DEGs were largely interferon-stimulated genes (ISGs), including *OAS1*, *MX1*, *IFI6*, and *XAF1* (Figure 7A). These ISG-high NCB patients showed minimal INCITE-reactivity during the ICI-4W window, with all three having among the four lowest INCITE upregulation scores (Figure S2B). T cell-specific ISG expression could therefore represent an intratumoral immune state that is not primed to generate effective anti-tumor immune responses to ICI+CTX. Similarly to *IGFBP2*, some ISGs showed different expression patterns in lymphocytes than in other compartments, which could prevent the detection of this phenomenon in bulk transcriptomics (Figure 7C). In this cohort, integrative scRNA-seq approaches, including both deconvolution and cell-type specific expression, showed significant power to reveal additional useful biomarkers.

## Discussion

We report here a comprehensive biomarker discovery study based on the uniquely designed phase I/II LUD2015-005 study, which treated 38 inoperable esophageal cancer patients with ICI alone for four weeks prior to ICI+CTX. Results showed that monitoring on-treatment changes in a T cell inflammation signature (INCITE) during this ICI-4W window can help assess ICI sensitivity. We also identified TMC as an independent but complementary biomarker to TMB for the prediction of ICI+CTX outcomes.

The LUD2015-005 ICI-4W window served as a proof-of-concept that four weeks of first-line ICI treatment is sufficient to induce anti-tumoral T cell responses in EAC, and afforded a unique opportunity to study ICI responses without confounding CTX. Early on-treatment changes associated with ICI response can be measured by INCITE identified in this study. INCITE upregulation during the first four weeks of ICI was also associated with overall ICI outcomes in an independent melanoma cohort,^41^ suggesting that INCITE upregulation has utility beyond EAC.

LUD2015-005 did not have a control group with ICI+CTX initiated concurrently. Future studies are needed to compare the magnitude of response to ICI-4W followed by ICI+CTX versus simultaneous ICI+CTX onset. Nevertheless, our results suggest that a short ICI-only window is sufficient and potentially desirable to prime anti-tumoral immune responses. Employing an initial ICI-only window could protect healthy immune cells from the negative aspects of CTX during an important period at ICI onset where anti-tumoral immune responses are augmented. Many CTX agents cause neutropenia and other forms of myelodepletion,^50^^,^^51^ but they can also deplete and impair the functionality of B cells and T cells,^52^^,^^53^^,^^54^^,^^55^ possibly limiting the full potential of adaptive immune responses to ICI. Initial ICI-only provides an opportunity to induce anti-tumoral immune responses in ICI-sensitive patients using the full complement of the immune system, potentially generating stronger anti-tumoral responses that could better withstand the negative effects of CTX. Additionally, an initial ICI-only window provides an opportunity to assess whether a patient’s immune system is fit-for-purpose to respond to ICI. Given the use of ICI+CTX in gastro-esophageal cancer and expanding interest in ICI-only for selected patients,^29^^,^^56^ this early assessment of ICI-only efficacy could be important to rapidly identify ICI-resistant tumors to consider for alternative therapies.

PD-L1 expression and TMB are commonly assessed ICI biomarkers. Histological PD-L1 CPS, a quantification method commonly used in clinics, was not assessed in LUD2015-005 due to sample limitations. While the RNA-seq PD-L1 quantification method employed here is highly correlated with histological methods in other settings,^57^ future CPS studies are needed to fully understand the utility of PD-L1 for αPD-L1 ICI in EAC. TMB quantification is FDA-approved as a companion diagnostic marker for ICI; however, questions remain concerning whether TMB is equally useful across cancer types.^4^ In gastro-esophageal cancers, TMB shows varying utility for ICI-containing regimens,^24^^,^^25^^,^^26^^,^^27^ emphasizing the need to discover additional biomarkers that could complement this biomarker. High TMB was associated with better OS in LUD2015-005, and its predictive power was significantly enhanced when combined with TMC.

TMC-high patients included all those attaining OS > 18 months in this study, suggesting TMC could be a particularly useful marker of durable benefit on ICI+CTX. While TMC is an NGS-derived estimate of monocyte content, validation experiments confirmed TMC was significantly associated with ground-truth monocyte RNA levels content. To facilitate wider use of TMB+TMC, future studies should adapt TMC assessment to targeted sequencing or histological assays, analogously to TMB quantitation through targeted sequencing panels rather than whole-exome sequencing.^58^ The finding that TMB and TMC predict outcomes for ICI-containing regimens but not for CTX-treated advanced EACs from TCGA and ICGC makes this biomarker combination an ideal tool to select EAC patients most likely to benefit from the addition of ICI to conventional CTX. While CTX largely targets cancer cells directly, ICI+CTX targets both tumor and immune cells in the tumor milieu. Therefore, the utility of TMB+TMC in predicting ICI+CTX outcomes may derive from the ability of this biomarker pair to simultaneously evaluate features of both cancer cells and their associated microenvironment that are likely to respond well to treatment.

TMC shows promising predictive utility for ICI-based therapies in both EBV-/MSS EAC and GC, but not for EBV+/MSI GC. This highlights the importance of molecular subtyping in biomarker discovery, particularly for GC. Previous studies identified four molecular GC subtypes: EBV-associated (EBV), microsatellite instability (MSI), chromosomal instability (CIN), and genomic stability (GS). EAC tumors have high molecular similarity to the CIN subtype, the most prevalent form of GC, but are distinct from EBV and MSI subtypes, which are rare or absent in EAC.^30^ A previous study of GC patients receiving ICI as second- or third-line therapy, which included EBV and MSI tumors, identified *CXCL11* and PD-L1 as strongly associated with ICI response.^28^^,^^44^ This contrasts with the present work in EAC, where neither PD-L1 nor T cell inflammation was predictive of outcomes, and the predominant transcriptional biomarker was TMC. As noted by the original authors, ICI response rates in this gastric cohort were much higher in EBV and MSI GCs (among those with available exome sequencing, 100% and 100%) compared with EBV-/MSS subtypes (GS: 12%, CIN: 5%).^28^ This suggests that, in unselected GC cohorts, biomarkers predicting ICI response are likely to be driven largely by responders with EBV and MSI-subtype tumors, potentially explaining why these results would not be shared by EAC cohorts. The higher response rates in EBV+/MSI GC than other subtypes, the high molecular similarity between CIN GC and EAC, and the similar predictive utility of TMC for our EAC cohort and for EBV-/MSS (but not EBV+/MSI) GC all suggest that EBV-/MSS GC subtypes behave more analogously to EAC with respect to ICI than to EBV+/MSI GC subtypes.

While some myeloid subsets have immunosuppressive functions, mature myeloid-derived effectors are also key mediators of phagocytosis, antigen presentation, and antibody-dependent cellular cytotoxicity in tumors.^59^^,^^60^^,^^61^^,^^62^^,^^63^^,^^64^ Inflammatory intratumoral macrophages (high CD68^+^/CD163^+^ ratio) and other inflammatory tumor-associated myeloid cells have been associated with improved ICI response.^59^^,^^65^ A transcriptional signature of tissue-resident macrophages was also shown to predict aPD-1 ICI response across melanoma, lung, and breast cancers.^66^ Nevertheless, the identification of monocyte-associated signatures, rather than those of differentiated inflammatory macrophages, as a strong ICI+CTX-specific predictive biomarker is a more novel finding.

Although PreTx TMC was significantly associated with CB in this cohort, the decrease in median monocyte content at ICI-4W in CB patients suggested that monocytes were unlikely to generate improved outcomes by directly carrying out sustained anti-tumor activity; rather, ICI may drive differentiation of intratumoral monocytes into pro-inflammatory myeloid effectors responsible for this association with improved outcomes. While some differentiated myeloid cells in solid tissues are long-lived tissue-resident populations from alternative progenitors, circulating monocytes are also recruited into tumors where they can differentiate into various intratumoral macrophage and DC subsets.^67^^,^^68^^,^^69^ Monocyte-derived TAMs can exist across a continuum of M1-like and M2-like states.^69^ While intratumoral monocyte-derived DCs predominantly resemble tissue-resident cDC2s,^70^ other populations, including mature LAMP3-high DCs arising in response to inflammatory stimuli,^71^^,^^72^ can also derive from monocytes.^73^^,^^74^ Taken together with our trajectory analysis, it appears likely that monocytes serve as an intratumoral myeloid progenitor pool, which could be polarized by ICI toward inflammatory myeloid effectors, thereby driving the association of TMC with ICI-induced tumor shrinkage and improved long-term ICI+CTX outcomes.

Indeed, while TMC-low tumors retained a TAM/M2-like myeloid phenotype at ICI-4W, ICI generated a more pro-inflammatory myeloid phenotype in TMC-high tumors, with a greater shift toward M1-like macrophages, cDC1s, cDC2s, and mature LAMP3-high DCs at ICI-4W. These differentiated effectors have various features likely to enhance anti-tumor responses. M1-polarized macrophages are better able to lyse internalized cells following phagocytosis, contributing to improved tumor control.^75^^,^^76^ M1-like macrophages in the LUD2015-005 atlas also showed increased expression of secreted proteins which can amplify anti-tumoral inflammatory responses, including *IL1A/IL1B* and *TNF*. The listed DC subsets help activate T cell responses through antigen presentation and costimulatory signals. *XCR1*+ cDC1s play a key role in cross-presenting tumor antigens on MHC I to activate antitumoral cytotoxic T cells,^77^^,^^78^ and mature LAMP3-high DCs in our dataset had the highest expression of CD80/CD86 costimulatory signals needed to fully activate T cell responses. cDC2 cells are phenotypically and functionally heterogeneous but also play an important role in directing CD4 responses in tumors.^78^^,^^79^ Therefore, the ICI-induced myeloid phenotype shift in TMC-high tumors can amplify anti-tumoral responses to therapy through both direct and T cell-mediated mechanisms. The inflammatory myeloid effectors generated by ICI in TMC-high patients likely act together with ICI-induced lymphocyte infiltration to drive the improved ICI-4W tumor shrinkage and overall ICI+CTX outcomes observed here.

Finally, we show the power of scRNA-seq to identify cell type-specific predictive biomarkers. Although patient numbers are limited (n = 8), the specificity of scRNA-seq enabled us to uncover DEGs that would be masked in bulk transcriptomics due to varying expression patterns across cellular compartments. In PreTx EAC cells, *IGFBP2* was most significantly associated with CB. Tumor-specific expression of *IGFBP2* in melanoma is associated with an ICI-favorable immune environment,^80^ showing this method can successfully uncover meaningful biological signals for cell type-specific expression patterns. In T cells, high PreTx ISG expression was found in NCB patients lacking inflammatory responses to ICI (INCITE-quiescence). While ISGs can mediate immune responses, their aberrant overexpression in T cells is linked with T cell dysfunction and death during severe viral infection, including HIV and SARS-COV-2, mediated through ISGs including the pro-apoptotic *XAF1*.^81^^,^^82^ Therefore, T cell-specific ISG expression could serve as a marker of dysfunctional or apoptosis-prone T cells unlikely to respond to ICI, agreeing with the INCITE-quiescence seen in these patients. These findings need further validation but show how scRNA-seq can reveal additional predictive biomarkers and biological insight.

These timely findings extend our understanding of ICI response in EAC. TMC and TMB mark patients likely to benefit from the addition of ICI to CTX in this setting and could help inform patient selection strategies for EAC and the growing list of other cancers treated by ICI+CTX.

## STAR★Methods

### Key resources table

**Table:** 

REAGENT or RESOURCE	SOURCE	IDENTIFIER
**Antibodies**		

Durvalumab	AstraZeneca UK, Ltd	RRID: AB_2616906
Tremelimumab	AstraZeneca UK, Ltd	N/A
CD14-FITC clone M5E2	Biolegend	RRID: AB_2616906
CD16-APC clone 3G8	Biolegend	RRID: AB_2616904
CD33-PE clone WM53	Biolegend	RRID: AB_2888908
CD56-BV510 clone 5.1H11	Biolegend	RRID: AB_2565632

**Biological samples**		

Biopsies of tumor and normal upper gastrointestinal tract tissues from esophageal cancer patients	LUD2015-005 clinical trial	NCT02735239, EudraCT 2015-005298-19
Biopsies of Barrett’s and normal esophagus from Barrett’s esophagus patients	University of Oxford, Translational Gastroenterology Unit Biobank, John Radcliffe Hospital	REC reference: 11/YH/0020

**Chemicals, peptides, and recombinant proteins**		

Heat-inactivated human serum	Sigma	Cat# H3667
DMEM/F-12	Gibco	Cat# 11330032
Red Blood Cell Lysis Solution (10×)	Miltenyi	Cat# 130-094-183
Bovine Serum Albumin Fraction V	Apollo Scientific	Cat# BIA3981
Acid-Phenol:Chloroform, pH 4.5 (with IAA, 125:24:1)	Invitrogen	Cat# AM9720
TURBO DNA-free Kit	ThermoFisher	Cat# AM1907
Animal Free Collagenase/Dispase Blend II	Sigma-Aldrich	Cat# SCR140
DNAse I	Sigma-Aldrich	Cat# D5025
Collagenase D	Sigma-Aldrich	Cat# 11088866001
Liberase DL	Sigma-Aldrich	Cat# 5466202001
Oxaliplatin	Eloxatin	N/A
Capecitabine	Xeloda	N/A
Zombie NIR	Biolegend	Cat# 423105

**Critical commercial assays**		

Chromium Single Cell Immune Profiling Assay (v1.1)	10x Genomics	https://www.10xgenomics.com/products/single-cell-immune-profiling
Dead Cell Removal Kit	Miltenyi	Cat# 130-090-101
mirVana miRNA Isolation Kit	ThermoFisher	Cat# AM1560
TruSeq Stranded Total RNA Library Prep Human/Mouse/Rat Kit	Illumina	Cat# 20020596
GeneJET Genomic DNA Purification Kit	ThermoFisher	Cat# K0722
Qubit RNA BR Assay	ThermoFisher	Cat# Q10211
Qubit dsDNA HS Assay	ThermoFisher	Cat# Q32851
Agilent RNA 6000 Pico Kit	Agilent	Cat# 5067-1513
Agilent High Sensitivity DNA Kit	Agilent	Cat# 5067-4626

**Deposited data**		

LUD2015-005 demographics and clinical outcomes (cut-off date 16 October 2020)	This paper	Table S8
LUD2015-005 bulk RNA-sequencing	This paper	EGA (EGAS00001006468)
LUD2015-005 single-cell RNA-sequencing	This paper	EGA (EGAS00001006469)
LUD2015-005 whole genome sequencing	This paper	EGA (EGAS00001006470)
LUD2015-005 monocyte spike-in bulk RNA-sequencing	This paper	EGA (EGAS00001007197)
The Cancer Genome Atlas (TCGA): ESCA cohort RNA-sequencing counts; mutational burden	Genomic Data Commons (GDC); TCGA Research Network, 2017	https://portal.gdc.cancer.gov/projects/TCGA-ESCA
International Cancer Genome Consortium: ESAD-UK cohort RNA-sequencing counts, mutational burden	Frankell et al.^31^	EGA (EGAD00001004423); https://dcc.icgc.org
Bulk RNA-sequencing data for normal esophagus, Barrett’s Esophagus (BE), and Esophageal Adenocarcinoma	Maag et al.^46^	ENA (PRJEB11797)
Bulk RNA-sequencing data for ICI-treated gastric cancer (Samsung cohort)	Kim et al.^28^	ENA (PRJEB25780)
Bulk RNA-sequencing data for ICI-treated gastric cancer (Yonsei and St. Mary’s cohorts)	Cheong et al.^47^	EGA (EGAD00001008091)
Bulk RNA-sequencing data for ICI-treated melanoma	Riaz et al.^41^	GEO (GSE91061)

**Software and algorithms**		

DESeq2 v1.30.1	Love et al.^84^	https://bioconductor.org/packages/release/bioc/html/DESeq2.html
FGSEA v1.17.1	Korotkevich et al.^85^	https://bioconductor.org/packages/release/bioc/html/fgsea.html
Cell Ranger v3.1.0	10x Genomics	https://support.10xgenomics.com/single-cell-gene-expression/software/downloads/3.1/
BayesPrism v2.0	Chu et al.^45^	https://github.com/Danko-Lab/TED/
Bisque v1.0.4	Jew et al.^86^	https://github.com/cozygene/bisque
BSEQ-sc v1.0	Baron et al.^87^	https://shenorrlab.github.io/bseqsc/index.html
MuSiC v0.1.1	Wang et al.^88^	https://xuranw.github.io/MuSiC/articles/MuSiC.html
CIBERSORTx (docker cibersortx/fractions:latest, created 2020-04-04)	Newman et al.^89^	https://cibersortx.stanford.edu/
Seurat v3.9.9.9038	Stuart et al.^90^	https://satijalab.org/seurat/index.html
sctransform v0.3.2.9002	Hafemeister and Satija,^91^	https://cran.r-project.org/package=sctransform
batchelor v1.2.4	Haghverdi et al.^92^	https://bioconductor.org/packages/release/bioc/html/batchelor.html
TCGAbiolinks v2.23.6	Colaprico et al.^93^	https://bioconductor.org/packages/release/bioc/html/TCGAbiolinks.html
survivalAnalysis v0.2.0	CRAN (Wiesweg)	https://cran.r-project.org/package=survivalAnalysis
SEER^∗^STAT v8.3.6	National Cancer Institute	https://seer.cancer.gov/seerstat/
MatchIt v4.3.3	Ho et al.^94^	https://github.com/kosukeimai/MatchIt
MSIsensor v1.2.0	Jia et al.^95^	https://github.com/xjtu-omics/msisensor-pro
R	R Core Team	https://www.r-project.org
pheatmap v1.0.12	CRAN (Kolde)	https://cran.r-project.org/package=pheatmap
ggplot2 v3.3.5	Wickham, 2016^96^	https://ggplot2.tidyverse.org/
Nextflow pipelines for WGS and bulk RNA-sequencing pre-processing and alignment, including references for software dependencies	This paper	https://doi.org/10.5281/zenodo.8003609 (archive of bitbucket.org/licroxford/carroll_etal_2023)
Reproducible code for downstream analysis and figure generation	This paper	https://doi.org/10.5281/zenodo.8003609 (archive of bitbucket.org/licroxford/carroll_etal_2023)

**Other**		

Flowmi Cell Strainers, 70uM for 1000uL pipette tips	Sigma-Aldrich	Cat# BAH136800070
CellTrics 100μM filter	Sysmex	Cat# 04-004-2328
Fisherbrand™ RNase-Free Disposable Pellet Pestles	Fisher Scientific	Cat# 13236679
AMPure XP	BeckmanCoulter	Cat# A63880
SPRIselect	Beckman Coulter	Cat# B23317

### Resource availability

#### Lead contact

Further information and requests for resources and reagents should be directed to and will be fulfilled by the lead contact, Xin Lu (xin.lu@ludwig.ox.ac.uk).

#### Materials availability

This study did not generate new unique reagents.

### Experimental model and subject details

#### LUD2015-005 inoperable cohort

The LUD2015-005 clinical trial is a phase I/II study of durvalumab as first-line therapy for patients with esophageal or gastro-esophageal cancer. Adult patients with a histologically confirmed diagnosis of locally advanced or metastatic esophageal or gastro-esophageal cancer were eligible for enrolment into the inoperable arms of the LUD2015-005 trial. Patients with previous systemic anti-cancer therapy for this same advanced disease were excluded from this study, as were patients who had been treated with ICI in any previous setting. Inclusion criteria included an Eastern Cooperative Oncology Group (ECOG) performance status of 0 or 1, and an anticipated lifespan of greater than 4 months.

Eligible patients were enrolled into one of three treatment arms for inoperable patients based on a dose escalation strategy, which proceeded to the next stage following establishment of acceptable safety profile at each step. Patients in the first stage (n = 12) received 750 mg of intravenous durvalumab administered biweekly (Q2W) as the ICI agent, those in the second (n = 5) received the same regimen of durvalumab plus a single 37.5 mg priming dose of tremelimumab, and those in the third (n = 21) received durvalumab plus a single 75 mg priming dose of tremelimumab. Expansion at the recommended dose (third stage) to at least 20 patients was driven by the need to establish the safety profile of combination therapy as well as a preliminary assessment of efficacy. The study was not designed to provide definitive information, but to deliver the basis for design and interpretation of future trials and to allow the interpretation of translational endpoints. After 4 weeks of treatment with these ICI agents alone, patients in all cohorts received a maximum of six cycles of chemotherapy with oxaliplatin and capecitabine, in addition to continued Q2W durvalumab. In the absence of a reason to discontinue treatment earlier, durvalumab infusions continued until the end of the final chemotherapy cycle. The demographic details of the 38 inoperable patients who received treatment on the LUD2015-005 trial can be found in Table S1.

#### Supplemental patient-derived material

For scRNA-sequencing, additional biopsies were also taken from three patients from the operable arms of the LUD2015-005 trial, which enrolled patients deemed suitable for surgery with curative intent. These operable EACs were treated in the neoadjuvant setting with a similar regimen of durvalumab, to which chemotherapy or chemoradiotherapy was added following an ICI-only window. Clinical and demographic details of these operable patients can be found in supplementary files at https://bitbucket.org/licroxford/carroll_etal_2023. Additional biopsies were also collected from patients undergoing routine monitoring of known BE by the Translational Gastroenterology Unit (TGU) Biobank (John Radcliffe Hospital; REC reference 11/YH/0020) and released to our team for analysis.

### Method details

#### Assessment of patient outcomes

The primary outcomes of this phase I/II study involved safety: the number of subjects reporting adverse events (up to one year after first dose of study medication), number of subjects experiencing a dose-limiting toxicity (up to 10 weeks after first dose of study medication), and any changes from treatment onset in laboratory evaluations (screening through three months after last dose of study medication). Secondary outcome measures were OS, PFS, and tumor response. Response was assessed using Immune-related Response Evaluation Criteria in Solid Tumors (irRECIST) criteria^32^ from CT scans conducted during the screening period and every 6 weeks thereafter during treatment. irRECIST outcomes were classified as complete responses (irCR), partial responses (irPR), progressive disease (irPD), or stable disease (irSD). One patient with an overall response of irNN, which occurs when there is no measurable target lesion at baseline and an absence of on-treatment irPD or irCR for non-target lesions, was considered to be irSD for the purposes of this work. Three patients who did not have an on-treatment CT scan for assessment of irRECIST criteria but were deemed to have clear on-treatment clinical progression were classified as irPD. irCR and irPR were classified as responders, and all other response categories were classified as non-responders. Tumor shrinkage during the ICI-4W window was calculated as the difference in sum of diameters of target lesions between pre-treatment and C1D8 CT scans. One patient (EAC-ECJA) did not have an available CT scan at the C1D8 timepoint, but did have an unscheduled CT scan just before (30 days after treatment onset), which was classified with C1D8 scans for the calculation of ICI-4W tumor shrinkage in this study.

Overall survival (OS) and progression free survival (PFS) were calculated including data from the post-study follow-up period (data cut-off 16 October 2020). A post-hoc outcome metric for long-term disease control was also defined, which was termed “clinical benefit”. A patient was deemed to have attained clinical benefit if a centralized review process determined they had 12 months of survival from the initiation of treatment without confirmed disease progression. One patient who received an alternative therapy within 12 months of treatment onset was excluded from clinical benefit analyses.

#### Research study procedures and sample collection

Endoscopic biopsies were collected for research analysis prior to the onset of treatment, during the fourth week of treatment with ICI alone (prior to the onset of chemotherapy), and at the end of the study treatment protocol (approximately 5–6 months after treatment onset). At each endoscopy, up to five 2 mm biopsy pairs were taken from the site of the tumor, while 1–2 pairs of normal control tissue were also taken from the descending duodenum (D2), gastric cardia (at least 2 cm distal to the gastro-esophageal junction (GEJ) or distal extent of lesion if crossing the GEJ), and normal esophagus (at least 2 cm proximal to the GEJ or proximal extent of lesion, whichever is more proximal). Each biopsy pair was split into two aliquots, one of which was immediately snap-frozen using dry ice or liquid nitrogen, while the other was slow frozen with 1 mL of fetal bovine serum (FBS) or heat-inactivated human serum with 10% DMSO and placed at −80°C in a controlled-rate freezing container. Snap and slow-frozen biopsies collected at local enrolment sites were stored at −80°C, and were shipped on dry ice to the central site for long-term storage at −80°C and subsequent analysis.

For supplemental operable EAC and Barrett’s patients profiled for scRNA-seq, a similar protocol was followed as for the LUD2015-005 inoperable cohort. For operable EAC patients, 2 mm endoscopic biopsies were obtained from tumor and normal esophagus prior to treatment and after 4 weeks of durvalumab alone, and were processed for snap and slow-freezing as above. At the surgery marking the end of the study protocol for these operable EAC patients, core biopsies of the tumor site and normal esophagus were also obtained from resected material.

For Barrett’s patients, 2mm endoscopic biopsies of Barrett’s Esophagus (BE) and paired normal esophagus (at least 2cm proximal to extent of BE) were collected at a single timepoint. Core biopsies from LUD2015-005 surgical specimens and the supplemental biopsies from BE patients were collected fresh in a 1.5 mL microcentrifuge tube containing DMEM/F-12 with 10% HS, and were kept on ice until processing. Supplemental biopsies were either slow-frozen in HS with 10% DMSO in a controlled-rate freezing jar as above or kept unfrozen on ice for dissociation prior to single-cell RNA-sequencing (see below).

#### Single-cell RNA-sequencing

##### Tissue dissociation

Prior to dissociation, slow-frozen biopsies were thawed by agitation of the cryovial in a 37°C water bath. For both fresh and frozen material, biopsies were washed in PBS, then transferred into either fresh (DMEM/F12 [Gibco] with 10% HS, 0.24 mg/mL Animal Free Collagenase/Dispase Blend II [Sigma], and 0.1 mg/mL DNAse I [Sigma]) or frozen tissue dissociation medium (DMEM with 10% FBS, 2.5 mg/mL Collagenase D [Sigma], 0.5 mg/mL Liberase DL [Sigma], 0.2 mg/mL DNAse I [Sigma]) and dissociated under constant rotation in an incubator at 37°C and 5% CO2 until fragments reduced considerably in size. The dissociated solution was then passed through a 100 μM CellTrics filter and washed through with quench buffer (PBS with 6% FBS), and the resulting filtrate was centrifuged to pellet cells. For fresh material, red blood cell lysis was performed using red blood cell lysis solution and dead cells were depleted using a magnetic-bead based approach. The resultant cell pellets were then resuspended in pre-chilled cell resuspension buffer (0.04% Bovine Serum Albumin in PBS), and filtered through a 70 μM Flowmi tip strainer. Filtered cell suspensions were then counted using an automated cell counter (Bio-Rad, TC20), diluted to a concentration of approximately 1 million total cells/mL in cell resuspension buffer, and kept on ice until encapsulation.

##### Encapsulation, library preparation, and sequencing

scRNA-seq was conducted using a 5′ scRNA-seq gene expression workflow (Chromium Single Cell Immune Profiling, Solution v1.1, 10x Genomics). Encapsulation of cells was performed using the Chromium Controller, and then GEM-RT, cDNA amplification, and construction of final libraries was conducted following manufacturer’s instructions. Size profiles of amplified cDNA and final sequencing-ready libraries was verified by on-chip electrophoresis (Agilent 2100 Bioanalyzer system) using the High Sensitivity DNA Kit, and concentration of final libraries was assessed by Qubit. Libraries were sequenced on an Illumina NovaSeq 6000 or NextSeq 500 (26 cycles read 1, 8 cycles i7 index, 98 cycles read 2), targeting a minimum of 20,000 reads per cell.

#### Bulk tissue RNA-sequencing

Total RNA was extracted from whole endoscopic biopsies using the mirVana miRNA Isolation Kit. Briefly, snap-frozen biopsies were transferred directly into pre-chilled lysis/binding buffer in a 1.5 mL microcentrifuge tube, and the tissue was homogenized on ice using a disposable RNAse-free pestle. Acid-Phenol:Chloroform RNA extraction was then performed following manufacturer’s instructions. Genomic DNA was digested from the eluted RNA using a 30-minute incubation with TURBO DNase. RNA concentration was then calculated using the Qubit RNA BR Assay and the RNA integrity number (RIN) was calculated using the Agilent 2100 Bioanalyzer system (Total RNA Assay). RNA was then plated and stored at −80°C. Prior to library preparation, thawed RNA was purified and concentrated using AMPure XP at a 2.8x ratio. Bead-based rRNA depletion and total RNA-sequencing library preparation with dual sample indexing was then conducted using the TruSeq Stranded Total RNA Library Prep Human/Mouse/Rat Kit. Libraries were sequenced on the Illumina HiSeq (75 cycles read 1, 8 cycles i7 index, 8 cycles i5 index, 75 cycles read 2) to a targeted depth of 50 million reads per library.

For the monocyte spike-in experiment, RNA extraction was conducted on additional esophageal cancer biopsies as above (3 EAC, 1 ESCC). RNA extraction from purified peripheral blood monocyte populations (see cell sorting) was conducted using the same protocol, with homogenization by vortexing rather than by pestle. For each of four tumor biopsies, subaliquots were generated with monocyte RNA spiked in at 0%, 0.5%, 1%, 2%, and 4% of the total RNA mass. For two patients with excess RNA, additional subaliquots with 0% and 8% monocyte RNA were generated. Library preparation was conducted on these RNA mixtures using the TruSeq Stranded Total RNA Library Prep Human/Mouse/Rat as above. All libraries were sequenced on the same NextSeq 2000 flow cell, targeting a depth of 50 million reads per sample.

#### Whole genome sequencing

Genomic DNA was extracted from whole endoscopic biopsies using GeneJET Genomic DNA Purification Kit according to manufacturer’s protocol. DNA quality and quantity was measured using Nanodrop and Qubit dsDNA HS assay kit. Extracted DNA samples were then sent out for library preparation using a PCR-free protocol and sequencing using the Illumina NovaSeq6000.

#### Cell sorting

PBMCs were isolated from healthy donors’ leukapheresis cones by density gradient separation (NHS Blood and Transplant, UK). Cells were resuspended in PBS containing 0.5% BSA (Sigma) and 2mM EDTA (Gibco), and stained with Zombie NIR, CD14-FITC clone M5E2, CD16-APC clone 3G8, CD33-PE clone WM53, and CD56-BV510 clone 5.1H11 (all from Biolegend). Non-classical and classical monocytes were sorted according to the gating strategy in Figure S7D using a BD FACSAria Fusion. To form the monocyte population for spike-in, sorted classical and non-classical monocytes were mixed in 10:1 ratio before RNA extraction, mirroring the proportions of intratumoral monocyte subpopulations in the LUD2015-005 scRNA-seq atlas.

#### Bioinformatics

##### Repository data access

For deconvolution, public bulk RNA-sequencing data for normal esophagus, BE, and EAC were retrieved from ENA accession PRJEB11797,^46^ and for ICI-treated GC from ENA accession PRJEB25780^28^ and EGA accession EGAD00001008091.^47^ Pre-processed RNA-seq counts and clinical outcome information for EAC patients in the TCGA were downloaded from the Genomic Data Commons using the TCGAbiolinks package^93^ in R. ICGC EAC raw sequencing and outcome data were downloaded from EGAD00001004423.^31^

##### Whole genome sequencing data analyses

Whole genome sequencing data were processed for alignment to the GRCh38 human genome, mutation calling, and copy number analysis in line with current best practices using a set of fully reproducible Nextflow^97^ pipelines. All pipeline definitions and associated information can be found at https://doi.org/10.5281/zenodo.8003609, an archive of the code repository at https://bitbucket.org/licroxford/carroll_etal_2023.

Briefly, mutations were called using a consensus of two of three callers (Strelka2, Mutect2 and Octopus). Following previous practice,^98^ tumor mutational burden was quantified as the rate of non-synonymous mutations in the coding space, reported as mutations per megabase. The size of the coding space used in this study was 35.6 Mb, calculated as the sum of the protein-coding exons from ENSEMBL100 gene annotation. Copy number variations were called using Battenberg. Ambiguities in calls of tumor ploidy were resolved by manual inspection and agreement of two authors (JK and IP). Using the majority clone number from Battenberg, Amplification was defined as >2 copy number-adjusted ploidy while deletions were defined as change of more than half of the baseline gene copies. For the TCGA cohort, tumor mutational burden metrics were downloaded from the source publication using TCGAbiolinks.^30^^,^^93^ For the ICGC cohort, mutation calls were downloaded from the ICGC data portal,^83^ and TMB was then calculated as the rate of non-synonymous coding variants per megabase as for LUD2015-005.

Genomic signatures of MSI were detected using MSIsensor.^95^ Tumors were determined to have MSI if the MSIsensor score was at least 3.5, a threshold which has previously been used to classify tumors as MSI using this tool.^99^^,^^100^

##### Single-cell RNA-sequencing data analyses

Processing of scRNA-seq data was conducted using the Cell Ranger pipeline from 10x Genomics. Briefly, raw BCL files were converted to demultiplexed FASTQ files using cellranger mkfastq with --use-bases-mask = Y26n^∗^,I8,Y98n^∗^. A custom reference sequence was then prepared using the concatenation of the GRCh38 human genome (ENSEMBL100) and supplementary contigs from the Genomic Data Commons alignment reference, namely the decoy contig hs38.d1 and a collection of viral genomes commonly found in human cancer samples. The full GENCODE v34 GTF was used as a gene annotation file, filtering out readthrough transcripts and annotations to PAR regions in chromosome Y as per Cell Ranger recommendations. A custom Cell Ranger reference set was then prepared from these sequence and annotation files using cellranger mkref with default arguments. Finally, the identification of true cells and construction of a UMI counts table for each sample was performed using cellranger count, and these per-sample counts were combined into a single matrix for downstream analysis using cellranger aggr without normalization.

Downstream analysis was conducted using Seurat.^90^ To begin, low-quality cells with less than 200 genes detected or more than 25% mitochondrial reads were removed. For initial clustering of the full dataset, we normalized using SCTransform,^91^ performed UMAP dimensionality reduction, and identified clusters with the default Louvain algorithm. Broad cell type partitions were then identified and subsetted into separate Seurat objects. To characterize smaller cell subsets, the above workflow was repeated on each of these partitioned objects. To minimize batch effect between fresh and frozen samples for these smaller objects, we conducted SCTransform normalization on fresh and frozen samples separately, and then integrated these two datasets using FastMNN,^92^ a mutual nearest neighbor batch correction method, before proceeding to dimensionality reduction. Marker genes for each identified cluster were calculated using the FindAllMarkers function, and these markers were used to manually assign cell type and cell subtype labels. Clusters representing low quality cells, detected based on abnormally high mitochondrial percentage, increased levels of a gene signature representative of dissociation-induced stress,^101^ and/or an abnormally low number of features within a given cell type, and clusters representing doublets, detected based on marker expression with an abnormally high number of features as supporting evidence, were identified and removed from the final scRNA-seq object. For detection of cell type-specific markers associated with patient outcome, a pseudobulk differential expression method was designed to minimize patient-specific genes and prioritize genes associated with outcome in multiple patients. Briefly, SCTransform-normalized counts from all cells in a given biopsy were summed, resulting in a pseudobulk sample. Differential expression was then conducted on these pseudobulk samples using DESeq2 as discussed below, with clinical benefit as the design formula. Figures visualizing the scRNA-seq findings were generated using a combination of Seurat and ggplot2.

##### Bulk RNA-sequencing analysis

Raw RNA-sequencing data were processed following best practices using a reproducible Nextflow^97^ pipeline. This workflow was based off of STAR^102^ alignment to the same reference used for scRNA-seq alignment, and generation of final counts matrices using featureCounts.^103^ The reproducible pipeline and associated information can be found at https://doi.org/10.5281/zenodo.8003609, an archive of the code repository at https://bitbucket.org/licroxford/carroll_etal_2023.

Differential expression was conducted using DESeq2 using a significance threshold of FDR <0.1 to identify differentially expressed genes. Default arguments were used, with the exception of the use of the “local” fit type to model dispersion estimates. Moderated gene-level log-fold changes were calculated within DESeq2 using the “ashr” method,^84^^,^^104^ and were used as the test statistic for gene set enrichment analysis, which was conducted using FGSEA^85^ on a database of pathways from Reactome and MSigDB (Hallmark and Gene Ontology). Log-normalized counts for heatmap visualization were generated using the varianceStabilizingTransformation function. To assess changes between timepoints while correcting for patient-specific effects, a design formula of ∼patient+timepoint was used.

##### Deconvolution analysis

45 of the 46 major cell types identified from scRNA-seq were used for bulk RNA-seq deconvolution, with one cell type (Schwann cells) excluded due to their rarity. For all deconvolution analyses, normalized counts in a linear scale were used for deconvolution, which were obtained for scRNA-seq data using the vst function from the sctransform package and for bulk RNA-seq data using the median of ratios method embedded in the estimateSizeFactors function from DESeq2. The combination of these normalization methods has been shown to provide accurate results across a variety of deconvolution approaches.^105^

For benchmarking, open-source tools Bisque,^86^ BSEQ-sc,^87^ MuSiC,^88^ and BayesPrism^45^ were installed from their GitHub repositories and ran on the same HPC cluster. We were unable to obtain source code for CIBERSORTx,^89^ and we therefore ran this tool from a docker container on an AWS instance. For all conditions tested, potentially confounding gene types (mitochondrial, ribosomal, sex-specific, and TCR/BCR variable region genes) were excluded from the input reference matrix prior to each run. Two input reference matrices were used, one including all genes, and another subsetted to just the 3000 most variable genes (as calculated by FindVariableFeatures from Seurat). For methods that allowed the sample or subject ID for each cell in the input reference matrix to be used during deconvolution (Bisque and MuSiC), the patient ID was passed to the deconvolution call. BayesPrism can additionally incorporate cell subtype information and malignant cell labels from the input reference matrix. Cell type and subtype labels were therefore used with all BayesPrism runs, with EAC given as the malignant cell type label for deconvolution of all gastro-esophageal adenocarcinoma samples, and ESCC for the deconvolution of squamous cell carcinoma samples (1 ESCC in monocyte spike-in experiment). For BSEQ-sc, markers of each cell type were pre-selected using Seurat’s FindAllMarkers and a downstream filtering strategy (pct.1 > 0.25, pct.2 < 0.75, p.adj <0.01). CIBERSORTx (cibersortx/fractions docker container) was run with 100 permutations for significance calculation and recommended settings for droplet-based scRNA-seq (--fraction 0 --rmbatchSmode TRUE).

Benchmarking was conducted on 80 pseudobulk samples, created by adding together scRNA-seq counts from known numbers of cells in the LUD2015-005 atlas. These pseudobulk samples were generated to represent a range of possible outcomes in this setting, including varying degrees of tumor purity, immune infiltration, and contamination with stroma and squamous epithelium. The Spearman correlation coefficient and normalized root mean square error (Normalized RMSE, defined as RMSE divided by the range of predicted values) were calculated, both against known RNA proportions (the fraction of counts assigned to each cell type) and known cell proportions (the cell numbers of each cell type used to generate the pseudobulk sample divided by the total number of cells). The same output of all algorithms was used for both RNA and cell comparisons, with the exception of BayesPrism; for this tool, the output of the main algorithm was used to predict RNA fraction, and the optional helper function estimate_sf was used to predict the cell fraction.

Final deconvolution analysis on bulk RNA-seq samples were conducted using BayesPrism. As restricting the input reference matrix to the subset of the 3000 most variable genes significantly improved the run time of these algorithms, while returning similar or even slightly improved accuracy (Figure S6 and Table S4), we used the 3000-gene matrix for BayesPrism deconvolution of these datasets. Potentially confounding genes were removed prior to the run, and EAC cells were labelled as malignant, as above. Normalized and scaled cell type proportion estimate for individual cell types were used to perform Cox regressions against overall survival, and estimates for cell types within broad cellular compartments were used to perform hierarchical clustering of samples (using the hclust method with “ward.D” linkage).

##### Survival analysis

The link between variables and survival outcomes were assessed using the survivalAnalysis package in R. analyze_survival was used to conduct univariate Cox regressions and to generate Kaplan-Meier plots, while analyze_multivariate was used for multivariate Cox regressions and forest plots. Unless otherwise noted, continuous variables were scaled and centered prior to regression. For propensity matching analysis, patient treatment, demographic, and clinical outcome data were extracted from the Surveillance, Epidemiology, and End Results Program (SEER),^33^ a US-based registry of disease outcomes, using SEER^∗^STAT. For comparison with inoperable LUD2015-005 patients, only patients with stage III or IV esophageal cancers treated with chemotherapy were included. Optimal matching between LUD2015-005 and this SEER subset was performed using the MatchIt package^94^ with optimal matching and a 1:4 ratio. Tumor stage, histological subtype, sex, age, and primary ethnicity were the covariates accounted for during propensity matching.

### Quantification and statistical analysis

All statistical analyses were done using R. Boxplot visualizations all have a vertical line denoting the minimum to maximum range (excluding outliers), the box marking Q1-Q3, and a horizontal line denoting median. Details for the statistical tests employed can be found in the relevant method details section and figure legends. In the case of multiple testing, p value adjustment was performed using the Benjamini-Hochberg procedure^106^ to control for the false discovery rate (FDR). A threshold of 0.05 was used to determine significance throughout, with the exception of DESeq2 differential expression testing, where the package default threshold of FDR<0.1 was used.

### Additional resources

The LUD2015-005 study is registered with ClinicalTrials.gov under NCT02735239 (https://clinicaltrials.gov/ct2/show/NCT02735239) and with EudraCT under 2015-005298-19 (https://www.clinicaltrialsregister.eu/ctr-search/trial/2015-005298-19/GB).

## Data Availability

Raw WGS, bulk RNA-seq, and scRNA-seq FASTQ files generated from LUD2015-005 patient samples have been deposited at the European Genome-phenome Archive (EGA), and accession numbers are listed in the key resources table. LUD2015-005 clinical outcome data can be found in Table S8. This paper also analyzes existing, publicly available datasets, whose accessions are also found in the key resources table. Nextflow pipelines for reproducing pre-processing workflows, as well as code and supplementary files for downstream analyses and figure generation, can be found at Zenodo: https://doi.org/10.5281/zenodo.8003609, an archive of the code repository at Bitbucket: https://bitbucket.org/licroxford/carroll_etal_2023.

## Supplemental information

Document S1. Figures S1–S8 and Tables S1 and S2

Table S3. Key differential expression and gene signature results from bulk RNA-sequencing, related to Figures 2, 3, S2, S3, and S4 Sheets 1–4: DESeq2 outputs with log fold change moderation (“ashr” method). Results are listed as follows: Categorical comparison of ICI-4W vs. PreTx EAC samples (Sheet 1, INCITE genes are labeled, positive log2FoldChange enriched in ICI-4W); all PreTx EAC samples, using the change in tumor size during ICI-4W as a continuous variable in the design formula (Sheet 2, negative log2FoldChange linked with tumor shrinkage); all ICI-4W EAC samples, using the change in tumor size during ICI-4W as a continuous variable in the design formula (Sheet 3, negative log2FoldChange linked with tumor shrinkage); categorical comparison of CB vs. NCB from PreTx EAC samples (Sheet 4, positive log2FoldChange enriched in CB). Significant DEGs (adjusted p value<0.1) are bolded. Sheet 5: correlation between the change in VST-normalized gene expression during the ICI-4W window (ICI-4W – PreTx values) and tumor shrinkage during the ICI-4W window (negative coefficients linked with tumor shrinkage). Pearson and Spearman correlation, p-value, and FDR are provided. Pearson coefficients were used as the GSEA statistic for Figure 2D, and both coefficients were used to generate Figure S2C-F. Sheet 6: PD-L1 (*CD274*) log10-transformed TPMs (0.1 offset) were Z-score normalized and subjected to Cox regression with OS and PFS, and summary statistics are provided. Sheet 7: Performance of INCITE-12 against other inflammatory gene signatures. Top: Correlation test p values (Pearson and Spearman) for the association between upregulation of listed gene sets during ICI-4W in LUD2015-005 (calculated as the sum of scaled VST-normalized counts) and target lesion shrinkage during ICI-4W, using same procedure as for INCITE-12 in Figure S2H. Middle: Significance of association between upregulation of listed gene sets and long-term ICI-only outcomes in a cohort of ipilimumab-naïve melanoma patients treated with αPD-1 ICI,^41^ as displayed in Figure S2J. p values are calculated by non-parametric tests (Kruskal-Wallis for 3-way response; Mann-Whitney U test for 2-way benefit comparison). Bottom: as above, but for a cohort of melanoma patients treated with αPD-1 ICI who had previously progressed on ipilimumab from the same publication.^41^

Table S4. Extended results of pseudobulk deconvolution algorithm benchmarking, related to STAR Methods Cell type-specific results for the benchmarking pseudobulk experiment shown in Figure S6 are provided for each of the five deconvolution algorithms in separate tabs. Results are shown for two input reference matrices: The full scRNA-seq reference matrix, and a reference matrix subset to just the 3000 most variable genes (using Seurat’s FindVariableFeatures). For each of these conditions, results of each algorithm were compared to both the known RNA proportion (i.e., the fraction of counts assigned to each cell type) and the known cell proportion (i.e., the number of cells from each cell type divided by the total number of cells) of each pseudobulk sample. Spearman correlation and normalized root mean square error (RMSE) (RMSE / range of predicted values) are shown. The run time of each algorithm using each input reference matrix is also displayed.

Table S5. LUD2015-005 deconvolution results and statistics, related to Figures 4 and 6 Sheet 1: The proportion of each of the 45 major cell types estimated by BayesPrism were used in Cox regressions with overall survival (OS). Results of each cell-type regression are provided from the PreTx timepoint. Sheet 2: ICI-4W cell composition of patients with TMC-high and TMC-low PreTx phenotypes. Patients were split into tumor monocyte content (TMC)-high and TMC-low based on the PreTx median of the subgroup of EAC patients with available PreTx and ICI-4W biopsies (n = 28). The value at ICI-4W for each cell type was compared between PreTx monocyte-high and PreTx monocyte-low patients. p values were calculated using a Mann-Whitney U-test. No significant results were found following FDR adjustment (FDR<0.1). Sheet 3: Full BayesPrism deconvolution output for all LUD2015-005 inoperable EAC biopsies (3000 gene reference matrix). Deconvolution results are reported as the proportion of RNA (out of 1) contributed by each cell type.

Table S6. Summary of reported pharmacological agents in TCGA-ESCA and ESAD-UK, related to Figures 4 and 5 Sheet 1: Pharmaceutical treatment information, available only for 43 of 185 cases in the TCGA-ESCA cohort, was downloaded from GDC. Drug names were cleaned and standardized, and are reported for all patients with available information. For each patient, a one represents that the drug was reported to be taken at least once, while a zero represents that the drug was not reported for that patient. The Total_Count column shows drug frequency across the TCGA-ESCA cohort. Sheet 2: Four fields reporting treatment agents are collated for each patient from specimen- and donor-level entries from the ICGC Data Portal.^83^ A merged summary field is provided below to summarize the treatment type(s) reported for each patient.

Table S7. Detailed results from whole-genome sequencing, related to Figure 5 Sheet 1: All nonsynonymous coding single-nucleotide variants (SNVs) and insertions or deletions (indels) found at the pre-treatment timepoint. The location (GRCh38 coordinates), reference and altered nucleotide base, gene containing the mutation, effect of the mutation, and the variant allele frequency (VAF) are shown. Location coordinates follow the browser extensible data (BED) format convention (zero-offset with a left-closed, right-open interval). Sheet 2: Details for all copy number aberrations detected in the major tumor clone identified in Battenberg analysis. The log R ratio (LogR) and B-allele frequency (BAF) are provided for each copy number variation (CNV) event. From the Battenberg analysis, the copy number of the major (nMajor) and minor (nMinor) alleles, as well as the cancer cell fraction (CCF) are provided for the major subclone. Column “dip.tetra” identifies the underlying genome ploidy (diploid or tetraploid). All genes falling in each CNV interval are identified. On both sheets, all aberrations involving putative EAC driver genes are noted in the rightmost column. For patients with WGS available from multiple PreTx biopsies, the hyphenated suffix in the sample column differentiates entries from different biopsies.

Table S8. Summary of patient metadata and key biomarker results, related to Figure 1, Table 1, and STAR Methods Patient demographics, treatment information, and clinical outcomes are displayed for each patient identifier, alongside selected biomarker results from this study. For pre-treatment values, tumor mutational burden is presented in coding mutations per Megabase, MSIsensor score reports the percentage of MSI somatic sites as reported by the MSIsensor algorithm, and tumor monocyte content is the raw output of BayesPrism (3,000 gene reference matrix) showing the fraction of tumor RNA predicted to be derived from monocytes. For metrics derived from changes during ICI-4W, INCITE change is the normalized average of change in all INCITE genes between PreTx and ICI-4W (scaled without centering, positive values represent upregulation at ICI-4W), while tumor shrinkage shows the change in target lesion burden between PreTx and C1D8 scans as a percentage of the PreTx value.

Document S2. Article plus supplemental information
