# Supplementary material for: Tumor monocyte content predicts immunochemotherapy outcomes in esophageal adenocarcinoma
Source: Cancer Cell. 2023 Jul 10;41(7):1222–1241.e7. doi: 10.1016/j.ccell.2023.06.006 (PMC11913779; doi:10.1016/j.ccell.2023.06.006)
Supplement: Document S1. Figures S1–S8 and Tables S1 and S2 [file mmc1.pdf]

Cancer Cell, Volume *41*

Supplemental information

Tumor monocyte content predicts immunochemotherapy outcomes in esophageal adenocarcinoma

Thomas M. Carroll, Joseph A. Chadwick, Richard P. Owen, Michael J. White, Joseph Kaplinsky, Iliana Peneva, Anna Frangou, Phil F. Xie, Jaeho Chang, Andrew Roth, Bob Amess, Sabrina A. James, Margarida Rei, Hannah S. Fuchs, Katy J. McCann, Ayo O. Omiyale, Brittany-Amber Jacobs, Simon R. Lord, Stewart Norris-Bulpitt, Sam T. Dobbie, Lucinda Griffiths, Kristen Aufiero Ramirez, Toni Ricciardi, Mary J. Macri, Aileen Ryan, Ralph R. Venhaus, Benoit J. Van den Eynde, Ioannis Karydis, Benjamin Schuster-Böckler, Mark R. Middleton, Xin Lu, and for the LUD2015-005 Project Team

SUPPLEMENTAL FIGURES

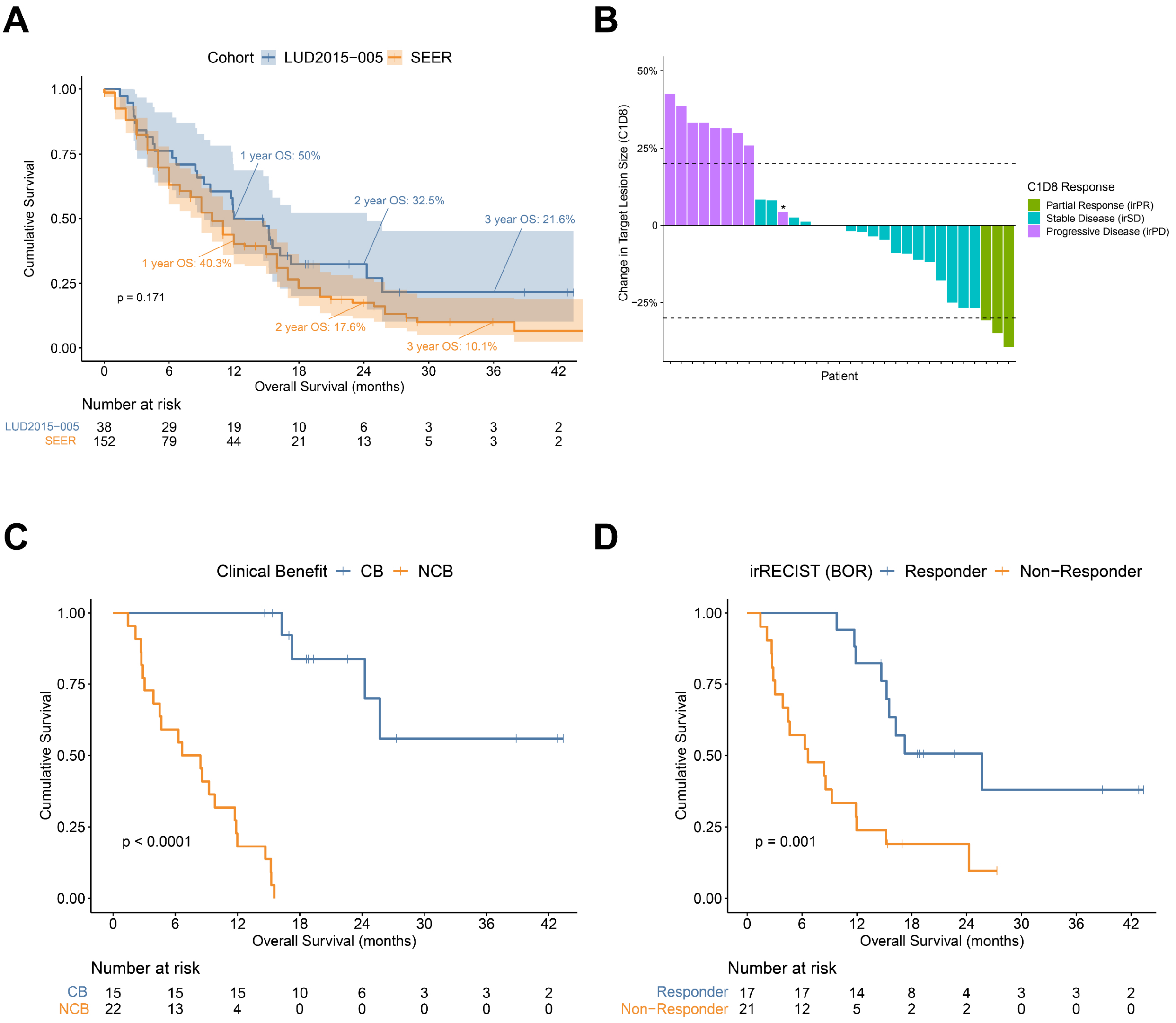

Figure S1 | Additional LUD2015-005 clinical analyses (related to Figure 1)

A. Propensity-matched cohort analysis between SEER[1] and LUD2015-005 inoperable esophageal cancer patients. SEER patients with stage III or IV EAC or ESCC with reported chemotherapy treatment since 2010 were selected for comparison. Propensity matching was conducted with optimal matching using a 1:4 ratio. The shaded region shows the 95% CI for each curve. Displayed p value was calculated by the log-rank test, testing the overall difference between the two groups. Listed OS percentages at fixed timepoints were extracted from the generated survival curve model using the survfit function from the survival package.

B. Waterfall plot of the shrinkage of target lesions at the C1D8 scan (sum of diameters; not including new or non-measurable lesions), approximately one week following the end of ICI-4W. A C1D8 scan with measurable target lesions was available for 31 of 38 treated patients. Each patient is colored by the irRECIST response classification at C1D8. An asterisk marks patients with irSD of target lesions but unequivocal progression (unconfirmed at this timepoint) due to new lesions.

C-D. Comparison of binary outcome classification systems and their link with long-term overall survival. Kaplan-Meier (KM) plots grouped by (**C**) clinical benefit (CB) and (**D**) irRECIST best overall response (BOR). Displayed p values were calculated using the log-rank method. One patient not assessable for CB was excluded from (**C**).

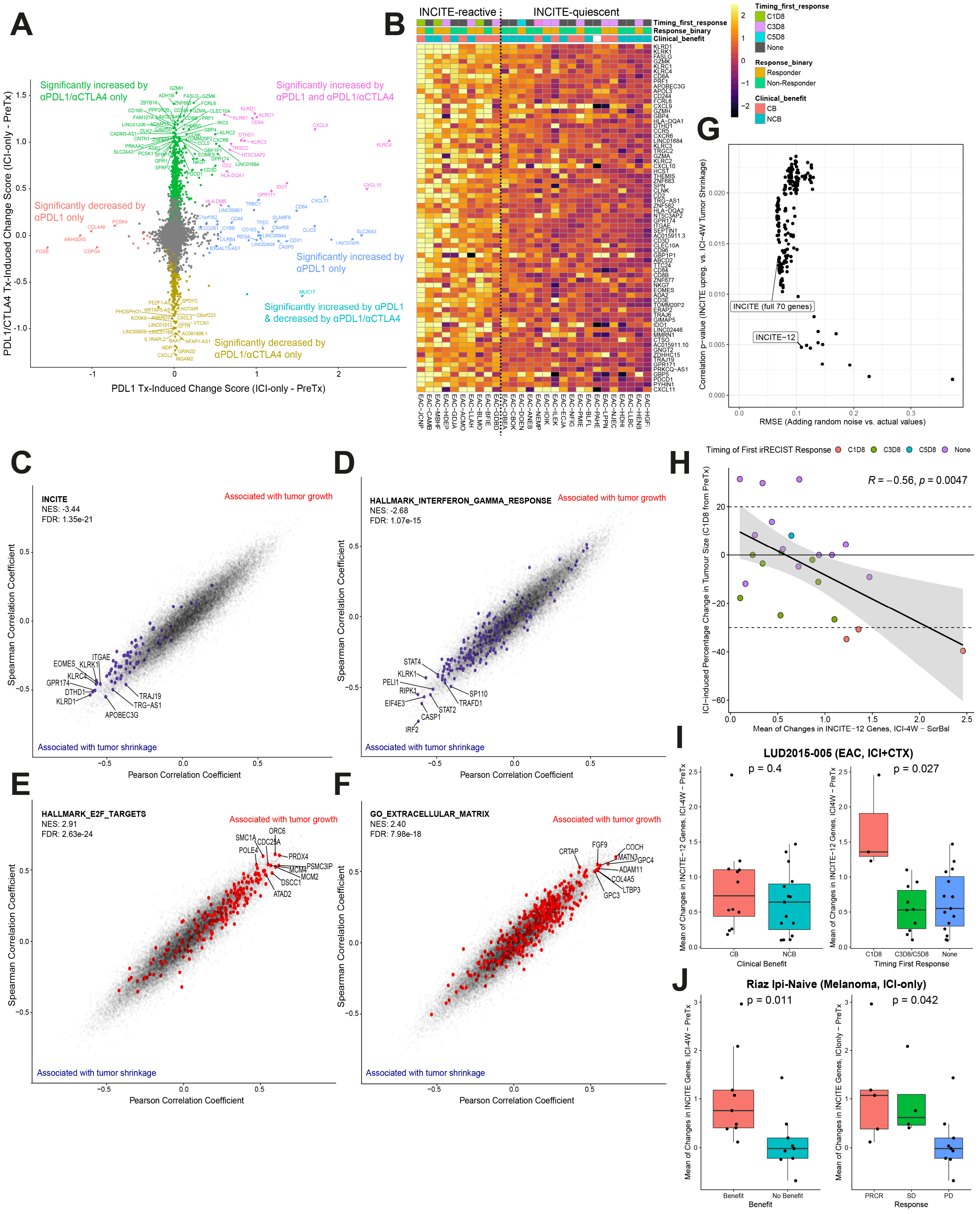
A
Significantly increased by αPDL1/αCTLA4 only
Significantly increased by αPDL1 and αPDL1/αCTLA4
Significantly decreased by αPDL1 only
Significantly increased by αPDL1 only
Significantly increased by αPDL1 & decreased by αPDL1/αCTLA4
Significantly decreased by αPDL1/αCTLA4 only
PDL1/CTLA4 Tx-Induced Change Score (ICI-only - PreTx)
PDL1 Tx-Induced Change Score (ICI-only - PreTx)
B
INCITE-reactive
INCITE-quiescent
Timing_first_response
Response_binary
Clinical_benefit
Timing_first_response
C1D8
C3D8
C5D8
None
Response_binary
Responder
Non-Responder
Clinical_benefit
CB
NCB
G
INCITE (full 70 genes)
INCITE−12
Correlation p-value (INCITE upreg. vs. ICI-4W Tumor Shrinkage)
RMSE (Adding random noise vs. actual values)
C
INCITE
NES: -3.44
FDR: 1.35e-21
Associated with tumor growth
Associated with tumor shrinkage
Spearman Correlation Coefficient
Pearson Correlation Coefficient
D
HALLMARK_INTERFERON_GAMMA_RESPONSE
NES: -2.68
FDR: 1.07e-15
Associated with tumor growth
Associated with tumor shrinkage
Spearman Correlation Coefficient
Pearson Correlation Coefficient
E
HALLMARK_E2F_TARGETS
NES: 2.91
FDR: 2.63e-24
Associated with tumor growth
Associated with tumor shrinkage
Spearman Correlation Coefficient
Pearson Correlation Coefficient
F
GO_EXTRACELLULAR_MATRIX
NES: 2.40
FDR: 7.98e-18
Associated with tumor growth
Associated with tumor shrinkage
Spearman Correlation Coefficient
Pearson Correlation Coefficient
H
Timing of First irRECIST Response
C1D8
C3D8
C5D8
None
R = −0.56, p = 0.0047
ICI−induced Percentage Change in Tumour Size (C1D8 from PreTx)
Mean of Changes in INCITE−12 Genes, ICI-4W − ScrBsl
I
LUD2015-005 (EAC, ICI+CTX)
p = 0.4
p = 0.027
Mean of Changes in INCITE−12 Genes, ICI-4W − PreTx
CB
NCB
Clinical Benefit
C1D8
C3D8/C5D8
None
Timing First Response
J
Riaz Ipi-Naive (Melanoma, ICI-only)
p = 0.011
p = 0.042
Mean of Changes in INCITE Genes, ICI-4W − PreTx
Mean of Changes in INCITE Genes, ICIonly − PreTx
Benefit
No Benefit
Benefit
PRCR
SD
PD
Response

Figure S2 | Dynamic differential expression analyses associated with ICI-4W tumor shrinkage (related to Figure 2)

A. Differential expression analysis for treatment-induced DEGs was conducted separately for αPD-L1 and αPD-L1+αCTLA-4 cohorts. Moderated LFCs are compared on x and y axes. Genes are colored based on the dataset(s) where they meet a 0.1 FDR significance threshold, as labelled.

B. Changes in INCITE genes were calculated as the difference between VST expression values at ICI-4W and PreTx for each patient. Patients were ordered according to the average magnitude in these VST expression changes, which were scaled without centering as in Figure 2C. The top 10 patients in terms of magnitude of INCITE upregulation were deemed “INCITE-reactive”. Hierarchical clustering was performed on rows (ward.D linkage).

C-F. Scatterplot of the Pearson and Spearman correlation coefficients between change in VST gene expression and change in target lesion size during the ICI-4W window. FGSEA summary statistics (NES and FDR) for the labelled pathway are reported as calculated in Figure 2D. Genes in the labelled pathway are colored in purple if the pathway is associated with tumor shrinkage, or red if associated with growth. Genes showing the most extreme Pearson and/or Spearman correlation coefficients in each pathway are labelled.

G. Identification of an optimal compact INCITE signature. Gene set sizes of the top ICI-upregulated genes, ranging in length for 1-100, were assessed for their correlation with tumor shrinkage during ICI-4W (p value calculated as in Figure 2C). To model how technical noise during measurement of each transcript may affect robustness of each signature size, a random value (sampled from a distribution with mean 0, sd = 0.5) was added to each measurement before calculating the same correlation. The x-axis represents the RMSE between real INCITE values and those calculated from this noise-modified dataset, with lower values representing less error.

H. As in Figure 2C, but calculated using INCITE-12 rather than the full INCITE signature (70 genes).

I. INCITE-12 upregulation during ICI-4W grouped according to CB category (left) and the timing at which an irRECIST response was first measured (right). C1D8 represents the scan immediately following the ICI-4W window, while C3D8/C5D8 are in the middle of the ICI+CTX window (see Figure 1A). p values calculated using non-parametric tests: Mann-Whitney U test for the 2-group comparison, and Kruskal-Wallis for the 3-group comparison.

J. INCITE-12 upregulation during an ICI-4W window in an ipilimumab-naïve melanoma cohort treated with αPD-1 ICI[2]. Results were grouped according to the main RECIST v1.1-based outcome classification systems used by the original authors: 2-group benefit (CR/PR/SD vs. PD, left) system (left) and 3-group response (CR/PR vs. SD vs. PD, right). p values calculated using non-parametric tests: Mann-Whitney U test for the 2-group comparison, and Kruskal-Wallis for the 3-group comparison.

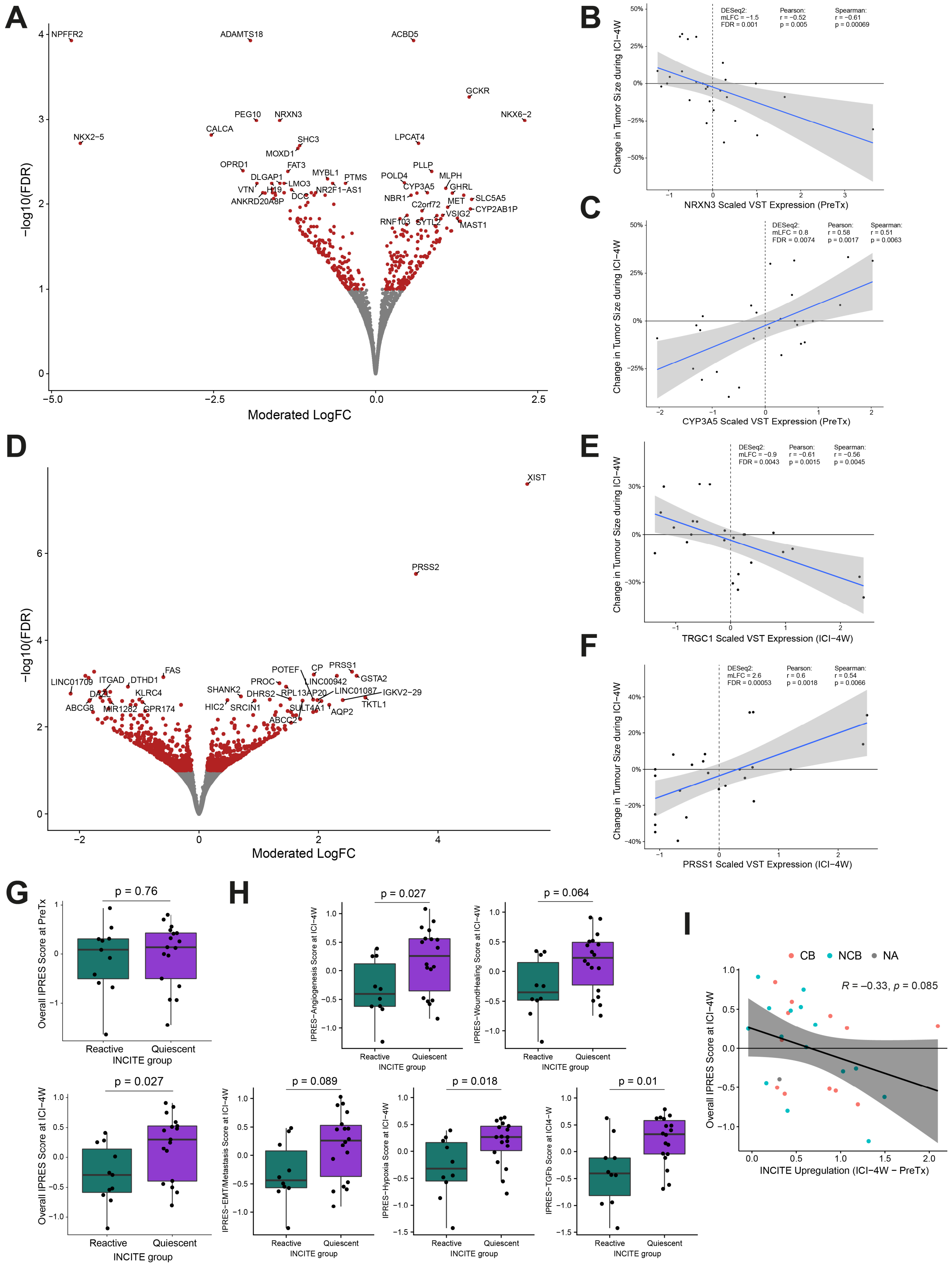
A
NPFFR2
ADAMTS18
ACBD5
GCKR
NKX6−2
PEG10
NRXN3
CALCA
NKX2−5
SHC3
LPCAT4
MOXD1
OPRD1
FAT3
PLLP
MYBL1
PTMS
POLD4
MLPH
DLGAP1
LMO3
CYP3A5
GHRL
VTN
H19
NR2F1−AS1
DCC
NBR1
MET
SLC5A5
ANKRD20A8P
C2orf72
CYP2AB1P
VSIG2
RNF103
SYTL2
MAST1
−log10(FDR)
Moderated LogFC
B
DESeq2: mLFC = −1.5 FDR = 0.001
Pearson: r = −0.52 p = 0.005
Spearman: r = −0.61 p = 0.00069
Change in Tumor Size during ICI−4W
NRXN3 Scaled VST Expression (PreTx)
C
DESeq2: mLFC = 0.8 FDR = 0.0074
Pearson: r = 0.58 p = 0.0017
Spearman: r = 0.51 p = 0.0063
Change in Tumor Size during ICI−4W
CYP3A5 Scaled VST Expression (PreTx)
D
XIST
PRSS2
FAS
LINC01709
ITGAD
DTHD1
DAZL
KLRC4
ABCG8
MIR1282
GPR174
POTEF
CP
PRSS1
GSTA2
PROC
LINC00942
SHANK2
DHRS2
RPL13AP20
LINC01087
IGKV2−29
TKTL1
HIC2
SRCIN1
SULT4A1
AQP2
ABCC2
−log10(FDR)
Moderated LogFC
E
DESeq2: mLFC = −0.9 FDR = 0.0043
Pearson: r = −0.61 p = 0.0015
Spearman: r = −0.56 p = 0.0045
Change in Tumour Size during ICI−4W
TRGC1 Scaled VST Expression (ICI−4W)
F
DESeq2: mLFC = 2.6 FDR = 0.00053
Pearson: r = 0.6 p = 0.0018
Spearman: r = 0.54 p = 0.0066
Change in Tumour Size during ICI−4W
PRSS1 Scaled VST Expression (ICI−4W)
G
p = 0.76
Overall IPRES Score at PreTx
Reactive
Quiescent
INCITE group
p = 0.027
Overall IPRES Score at ICI−4W
Reactive
Quiescent
INCITE group
H
p = 0.027
IPRES−Angiogenesis Score at ICI−4W
p = 0.064
IPRES−WoundHealing Score at ICI−4W
p = 0.089
IPRES−EMT/Metastasis Score at ICI−4W
p = 0.018
IPRES−Hypoxia Score at ICI−4W
p = 0.01
IPRES−TGFb Score at ICI4−W
Reactive
Quiescent
INCITE group
I
CB
NCB
NA
R = −0.33, p = 0.085
Overall IPRES Score at ICI−4W
INCITE Upregulation (ICI−4W − PreTx)

Figure S3 | Single-timepoint differential expression analyses associated with ICI-4W tumor shrinkage (related to Figure 2)

A. Volcano plot showing moderated LFCs and -log10(FDR) for a differential expression test comparing PreTx expression values and tumor size changes during ICI-4W as a numerical covariate. Points in red met the threshold for differential expression (FDR < 0.1).

B-C. Correlation plots between PreTx gene expression and tumor size changes during ICI-4W for example DEGs. Gene expression is presented as scaled and normalized VST-transformed counts. Summary statistics for Pearson and Spearman correlation tests are shown, as well as for the DESeq2 test from **A**.

D-F. As in **A-C**, but for ICI-4W timepoint.

G. IPRES scores (mean of Z-scored logTPMs across all INCITE genes) at ICI-4W grouped by INCITE upregulation classification (ICI-4W – PreTx). p value was calculated using a Mann-Whitney U test.

H. As in **G**, but IPRES scores were calculated separately for each of 5 major modules reported in Figure 2D of the source publication[3].

I. Comparison between INCITE upregulation (ICI-4W – PreTx) and overall IPRES score at ICI-4W, with points colored by clinical benefit grouping. Correlation coefficient and corresponding p value calculated using the Pearson method.

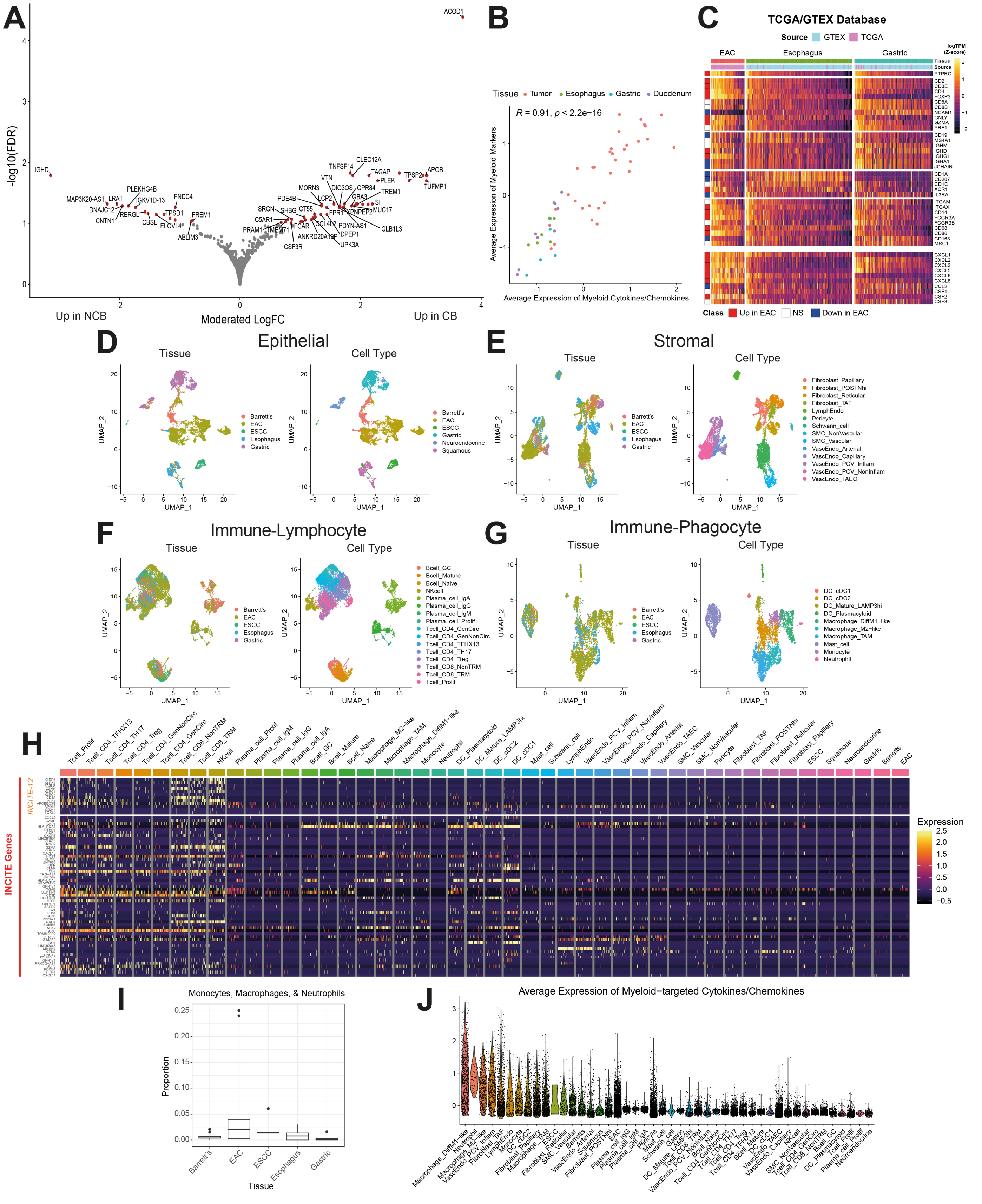

A
B
C
TCGA/GTEX Database
D
Epithelial
E
Stromal
F
Immune-Lymphocyte
G
Immune-Phagocyte
H
I
Monocytes, Macrophages, & Neutrophils
J
Average Expression of Myeloid−targeted Cytokines/Chemokines

Figure S4 | Clinical benefit volcano plot, TCGA/GTEX myeloid markers, and LUD2015-005 atlas plots (related to Figure 3)

A. Differential expression analysis between clinical benefit (CB) and no clinical benefit (NCB) Pre-Tx biopsies, showing the moderated log2 fold change (calculated within DESeq2 using the ashr method) and FDR (-log10 transformed). Significant DEGs (FDR < 0.1) were highlighted in red and labelled. Some non-coding genes without informative gene symbols were omitted from labelling.

B. Scatterplot showing the mean of logTPM Z-scores for the myeloid markers versus myeloid cytokines and chemokines (from Figure 3B) for each sample. The Pearson correlation coefficient and corresponding p value are shown.

C. Heatmap of Z-score normalized logTPM expression for immune markers and myeloid-targeted cytokines and chemokines as in Figure 3B, but for a validation cohort of EAC and normal GI samples from TCGA and GTEX. Genes that met the significance threshold (FDR < 0.1) were marked as significantly up or down in EAC.

D-G. UMAP visualizations colored by the tissue type (left) and identified cell type (right) for (**D**) Epithelium, (**E**) Stroma, (**F**) Immune-Lymphocyte, and (**G**) Immune-Phagocyte cellular compartments.

H. Heatmap showing the average expression (scaled, log-normalized counts) of INCITE genes detected in the LUD2015-005 scRNA-seq atlas, grouped by cell type. A maximum of 75 cells per cell type were selected using random sampling.

I. Boxplot of proportion of monocyte, macrophages, and neutrophils from scRNA-seq grouped by tissue type (PreTx samples only).

J. Violin plot for the Seurat-calculated module score of the panel of myeloid-targeted cytokines and chemokines shown in Figure 3B, grouped along the x-axis by cell subtype, with each point representing a single cell from EAC samples. Cell subtypes were arranged by decreasing mean expression of this panel.

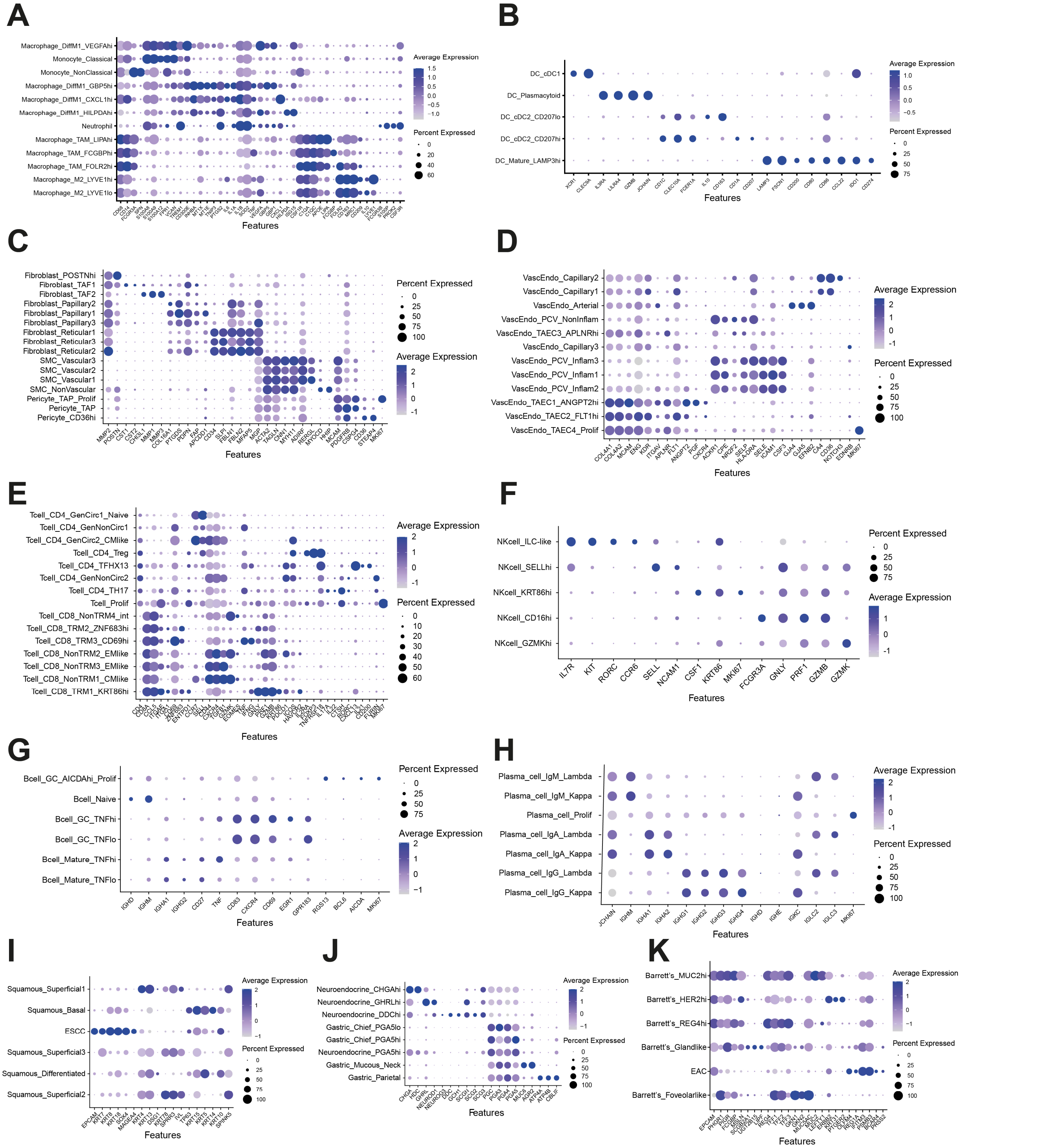

Figure S5 | Markers of identified cell types in the LUD2015-005 atlas (related to Figure 3)
Dot plots of selected markers that differentiate identified cell subtypes in (**A**) monocytes, macrophages, and neutrophils, (**B**) dendritic cells, (**C**) fibroblasts, (**D**) endothelial cells, (**E**) T cells, (**F**) NK cells, (**G**) B cells, (**H**) plasma cells, and (**I-K**) epithelium. For clarity, epithelial plots are split into (**I**) squamous epithelium, (**J**) gastric and neuro-endocrine cells, and (**K**) other non-squamous epithelium. The size of the dot represents the percent of cells within each column expressing the gene, while the color is the average expression level. Rows are ordered by hierarchical clustering.

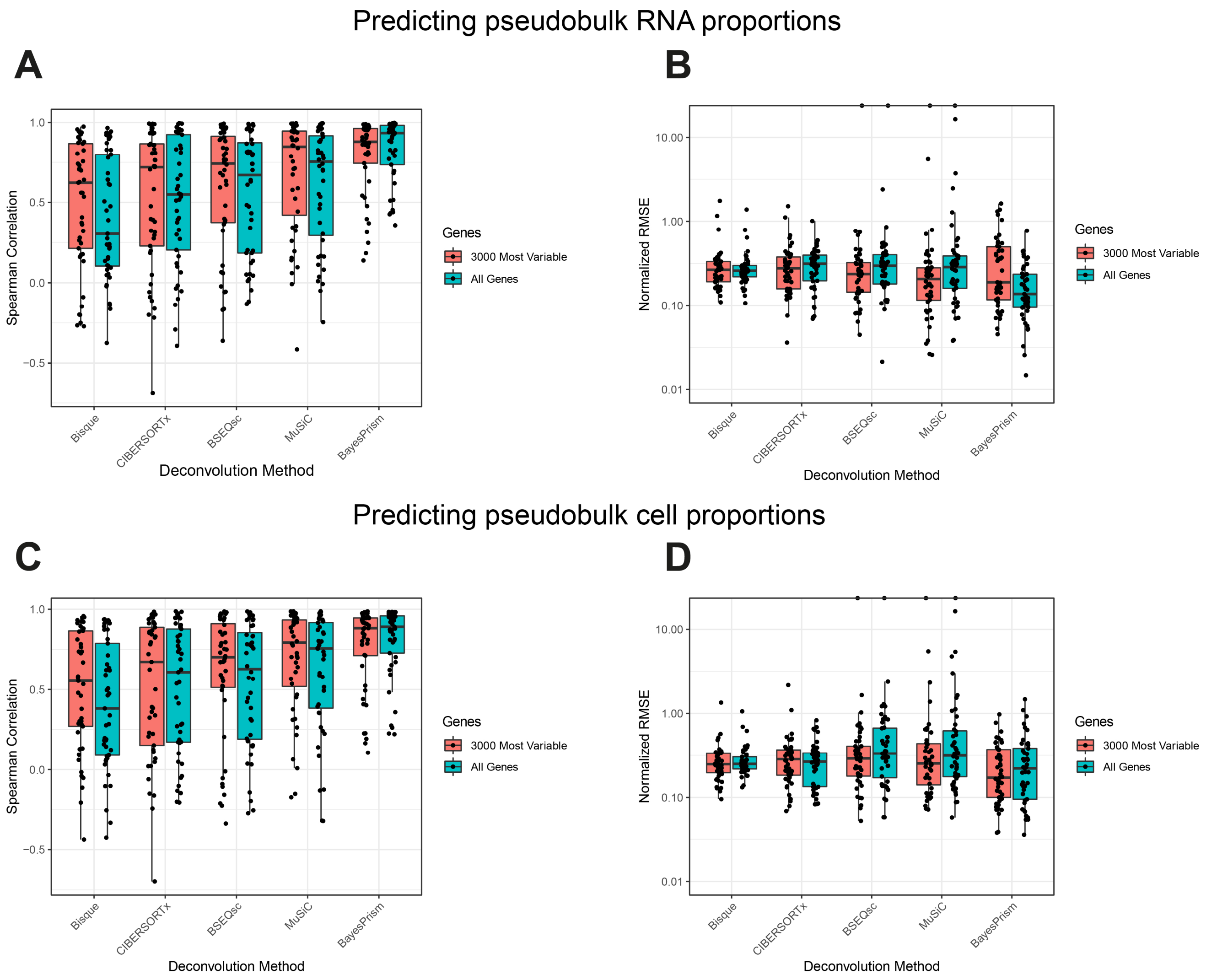

Figure S6 | Pseudobulk benchmarking of deconvolution algorithms (related to STAR Methods)

A-B. Estimates of 45 major cell types were generated from pseudobulk data for five deconvolution algorithms. Two scRNA-seq input reference matrices were assessed for all tools: One with all genes included, and the other with just the 3000 most variable genes (as determined using Seurat's FindVariableFeatures). Deconvolution estimates were compared with the known RNA fraction contributed by each cell type in the pseudobulk data (i.e. the number of counts assigned to a given cell type divided by the total number of counts). (**A**) Spearman correlation of the deconvolution estimates with the RNA fraction, with each dot representing one of the 45 cell types assessed by deconvolution. (**B**) is as in **A**, but for normalized root mean square error (RMSE), defined as the RMSE divided by the range of the predicted values.

C-D. As above, except deconvolution estimates were instead compared with the proportion of cells in each pseudobulk sample (i.e. the number of cells of a given type divided by the total number of cells) rather than total RNA proportion. (**C**) and (**D**) show Spearman correlation and normalized RMSE, respectively. The same main output of all algorithms was used for both RNA and cell comparisons, with the exception of BayesPrism; for this tool, the output of the main algorithm was used to predict RNA fraction, and the optional helper function estimate_sf was used to predict the cell fraction.

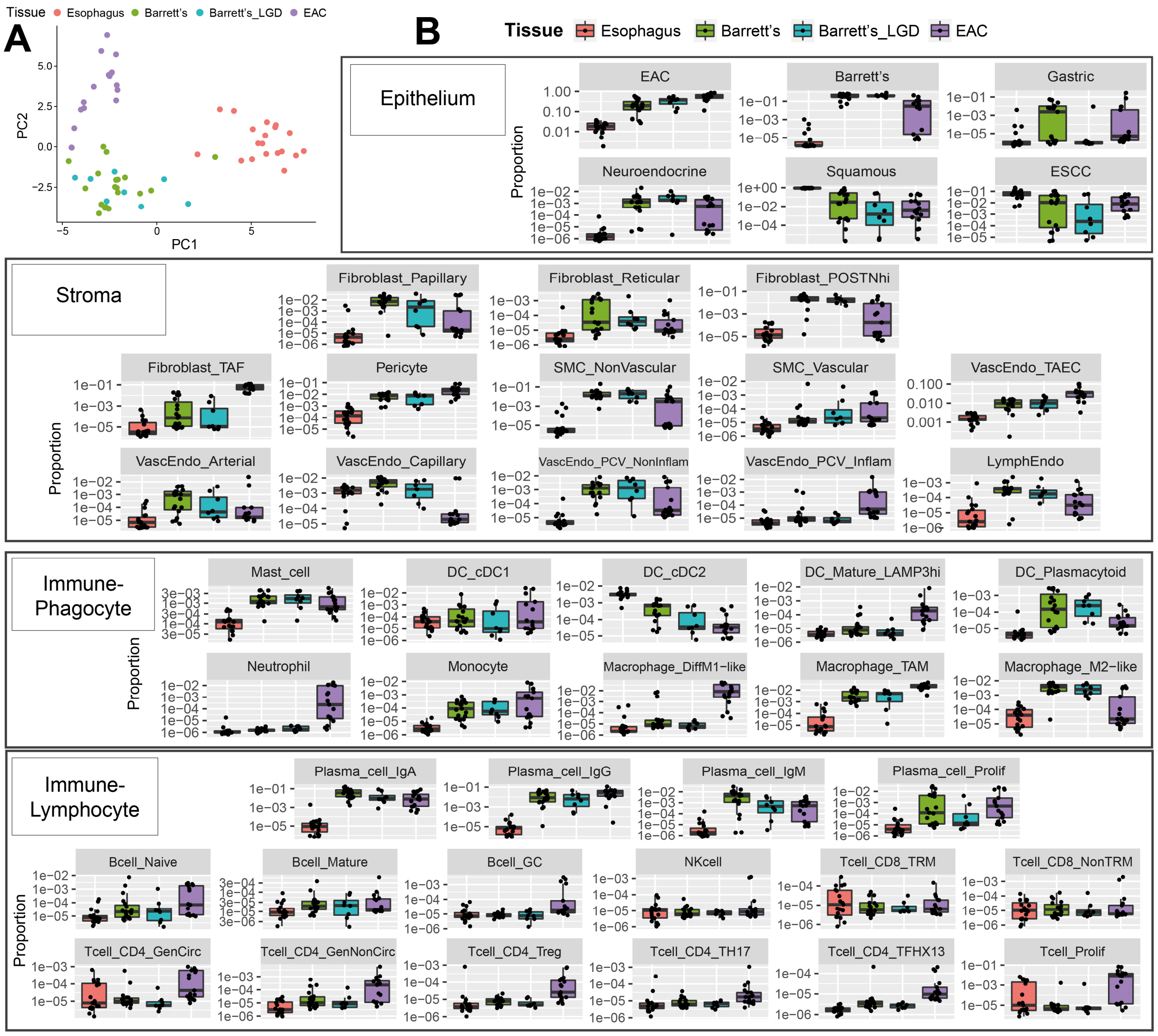

C

Estimated Proportion	Tissue Type: Esophagus (n = 19)	BE (n = 19)	BE with LGD (n = 8)	EAC (n = 17)
Squamous	**0.901**	0.172	0.064	0.050
BE	8.00E-05	**0.372**	**0.443**	0.076
EAC	0.019	0.230	0.346	**0.556**

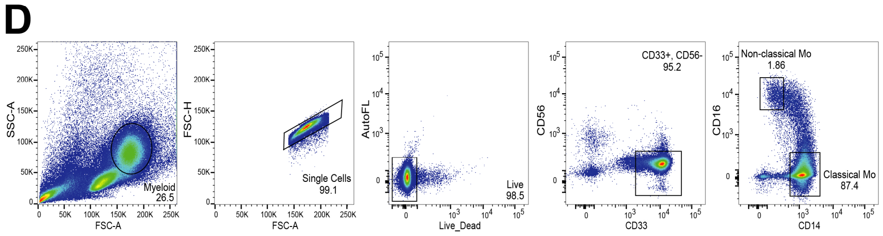

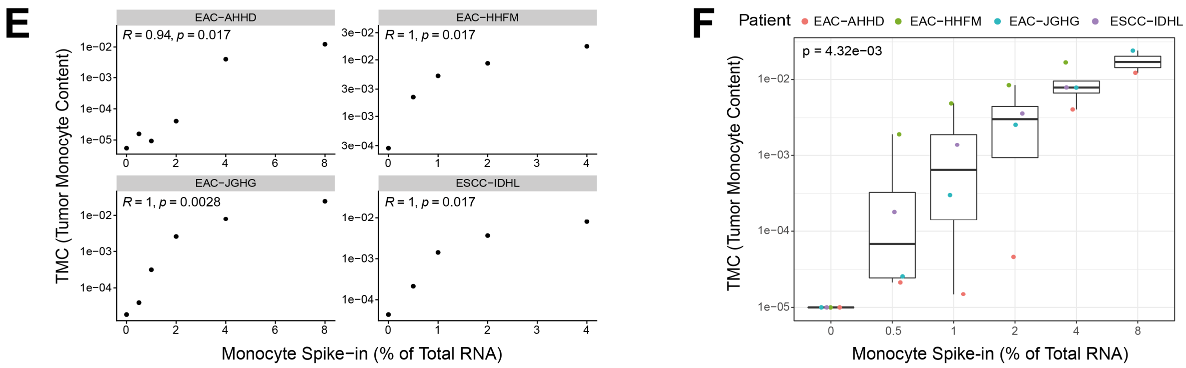

Figure S7 | Further validation of deconvolution and TMC estimates (related to Figure 4)

A-C. Validation of deconvolution performance using esophageal transcriptome data from Maag et al. (PRJEB11797)[4]. (**A**) Principal component analysis (PCA) on cell composition deconvolution estimates, colored by tissue of origin. Cell composition values were scaled and centered prior to PCA. (**B**) Boxplots showing the proportion of each cell type estimated by BayesPrism deconvolution for each of the four tissue types in this dataset: normal esophagus, Barrett's esophagus (BE), Barrett's esophagus with low-grade dysplasia (BE w/ LGD), and esophageal adenocarcinoma (EAC). (**C**) Mean deconvolution-assessed proportion of three epithelial cell types (rows) for each of these four esophageal tissue types (columns).

D. Gating strategy employed to purify monocyte populations for monocyte spike-in experiment. Labels indicate the gated population and the percentage of events falling in that gate. For the first four plots, the gated populations are used as parent populations for the subsequent plot. Sorted classical/non-classical monocytes populations are identified on the final rightmost plot based on CD14/CD16 staining. Monocyte RNA for spike-in experiments consisted of classical:non-classical monocyte RNA mixed at a 10:1 ratio.

E-F. Real-world validation of TMC using tumor RNA with known quantities of spiked-in monocyte RNA. For the patients with two replicates for the 0% spike-in level (EAC-AHHD and EAC-JGHG), TMC is presented as the mean of those two replicates for that level. (**E**) Comparison of deconvolution-assessed TMC and ground-truth monocyte spike-in levels (the percentage of purified monocyte RNA in the total library) for each individual patient. Correlation statistics were calculated using the Spearman method. (**F**) Distribution of TMC at each spike-in level grouped across patients. To correct for differing baseline TMC values for each tumor, the TMC value at 0% spike-in for each patient is subtracted from all TMC values for that patient. A pseudocount (1e-5) is added to allow log-scale plotting. Displayed p value calculated by the Kruskal-Wallis test shows a significant association between TMC and ground-truth monocyte content.

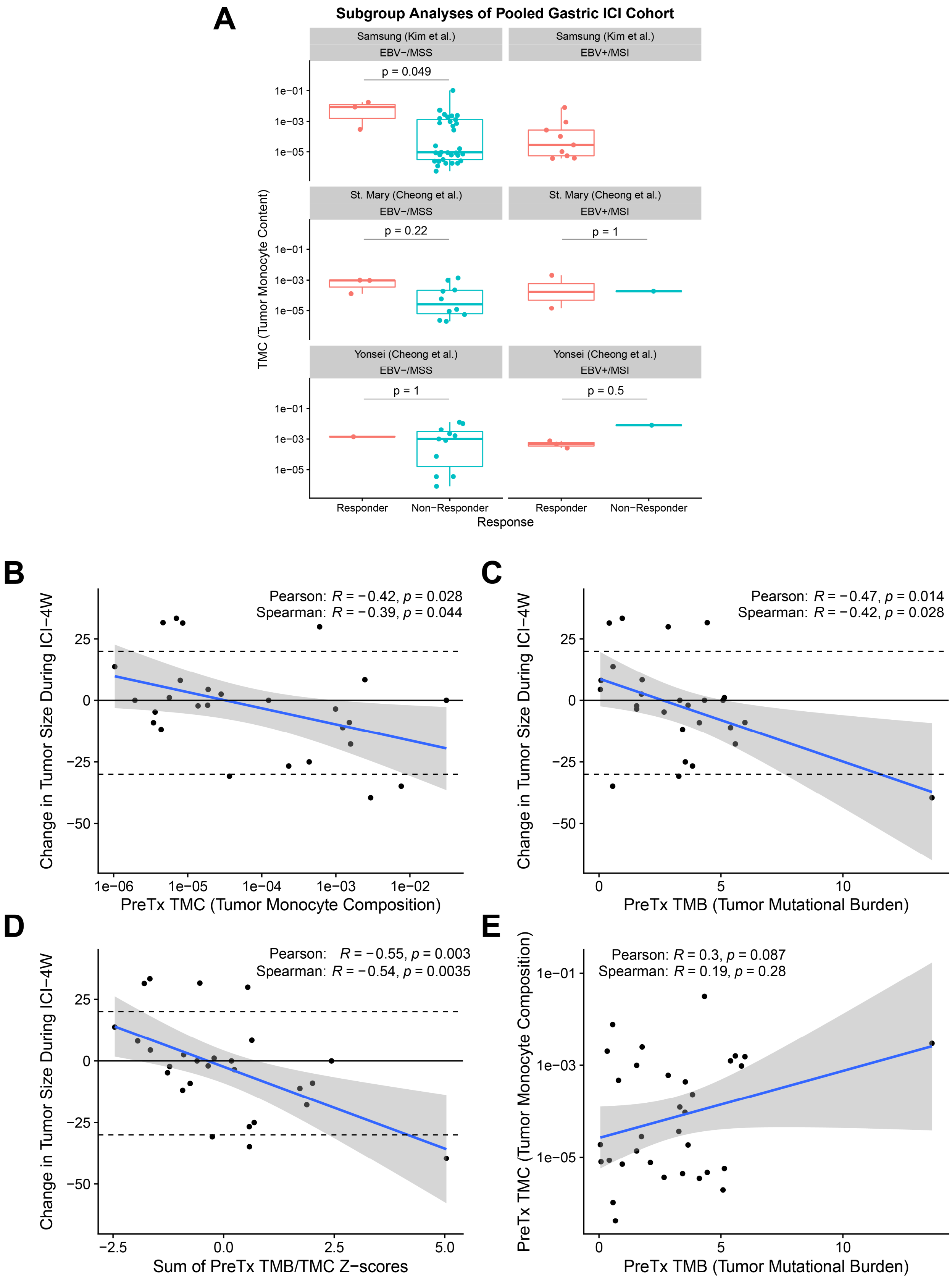
A
Subgroup Analyses of Pooled Gastric ICI Cohort
Samsung (Kim et al.) EBV−/MSS
Samsung (Kim et al.) EBV+/MSI
St. Mary (Cheong et al.) EBV−/MSS
St. Mary (Cheong et al.) EBV+/MSI
Yonsei (Cheong et al.) EBV−/MSS
Yonsei (Cheong et al.) EBV+/MSI
p = 0.049
p = 0.22
p = 1
p = 1
p = 0.5
TMC (Tumor Monocyte Content)
1e−01
1e−03
1e−05
Responder
Non−Responder
Response
B
Pearson: R = −0.42, p = 0.028
Spearman: R = −0.39, p = 0.044
Change in Tumor Size During ICI−4W
PreTx TMC (Tumor Monocyte Composition)
1e−06
1e−05
1e−04
1e−03
1e−02
C
Pearson: R = −0.47, p = 0.014
Spearman: R = −0.42, p = 0.028
Change in Tumor Size During ICI−4W
PreTx TMB (Tumor Mutational Burden)
D
Pearson: R = −0.55, p = 0.003
Spearman: R = −0.54, p = 0.0035
Change in Tumor Size During ICI−4W
Sum of PreTx TMB/TMC Z−scores
−2.5
0.0
2.5
5.0
E
Pearson: R = 0.3, p = 0.087
Spearman: R = 0.19, p = 0.28
PreTx TMC (Tumor Monocyte Composition)
PreTx TMB (Tumor Mutational Burden)
25
0
−25
−50
5
10

Figure S8 | Gastric cancer TMC subgroup analyses and relevance of TMB+TMC to ICI (related to Figures 4 and 5)

A. Subgroup analyses for the three centers comprising the pooled gastric ICI-treated cohort[5,6] in Figure 4E, showing a similar trend towards higher TMC values in ICI responders from EBV-/MSS tumors in all three subgroups. p values calculated using a Mann-Whitney U test.

B. Scatter plot of tumor shrinkage during ICI-4W (percentage change in target lesion size between PreTx and C1D8 CT scan as in Figure 2C, with positive values representing tumor growth) and pre-treatment tumor monocyte content (TMC). X-axis values are log10-transformed, and represent the estimated fraction of RNA in tumor biopsies derived from monocytes. p values and correlation coefficients calculated using the Pearson and Spearman methods are displayed. Dashed lines show the threshold for irRECIST progression (+20%, unconfirmed at the C1D8 scan) and response (-30%).

C. As in **B**, but the x-axis shows pre-treatment coding tumor mutational burden (TMB, presented in mutations per megabase).

D. As in **B-C**, but the x-axis is the sum of Z-scores for TMB and log10-transormed TMC.

E. Plot comparing the correlation of pre-treatment coding TMB with pre-treatment TMC (scale for TMC values is log10-transformed, as in **B**). p value and correlation coefficient calculated using the Pearson method.

SUPPLEMENTAL TABLES

ICI Agent	αPD-L1 (n=12)	αPD-L1+37.5mg αCTLA-4 (n=5)	αPD-L1+75mg αCTLA-4 (n=21)	Total (n=38)
Age at consent- years				
Median (range)	60 (23-75)	55 (29-67)	58 (42-78)	59 (23-78)
Sex- no. (%)				
Male	10 (83.3%)	4 (80%)	17 (81.0%)	31 (81.6%)
Female	2 (16.7%)	1 (20%)	4 (19.0%)	7 (18.4%)
Ethnicity- no. (%)				
White, British	10 (83.3%)	5 (100%)	20 (95.2%)	35 (92.1%)
White, Any other	2 (16.7%)	0	0	2 (5.3%)
Asian or Asian British	0	0	1 (4.8%)	1 (2.6%)
Stage at study entry- no. (%)				
IIIA	0	0	1 (4.8%)	1 (2.6%)
IIIB	1 (8.3%)	0	0	1 (2.6%)
IIIC	0	0	1 (4.8%)	1 (2.6%)
IV	11 (91.7%)	5 (100%)	19 (90.5%)	35 (92.1%)
ECOG Status- no. (%)				
0	12 (100%)	3 (60%)	13 (61.9%)	28 (73.7%)
1	0	2 (40%)	8 (38.1%)	10 (26.3%)

Table S1 | Clinical characteristics of the inoperable LUD2015-005 cohorts (related to Table 1)
The characteristics at enrolment of the 38 patients with inoperable esophageal cancer who received treatment on the LUD2015-005 study are presented, categorized by the ICI agent used. ECOG refers to the patient performance status: 0 is fully active, while 1 is ambulatory but restricted in physically strenuous activity.

Treatment-emergent adverse events in ITT population:

ICI Agent	αPD-L1 (n=12)	αPD-L1+37.5mg αCTLA-4 (n=5)	αPD-L1+75mg αCTLA-4 (n=21)	Total (n=38)
Reported TEAEs- no. (%)				
Any CTCAE grade	12 (100%)	5 (100%)	21 (100%)	38 (100%)
CTCAE Grades 3-5	11 (91.7%)	4 (80%)	14 (66.7%)	29 (76.3%)
Reported durvalumab-related TEAEs- no. (%)				
Any CTCAE grade	9 (75%)	5 (100%)	17 (81%)	31 (81.6%)
CTCAE Grades 3-5	3 (25%)	1 (20%)	7 (33.3%)	11 (29%)

DLTs encountered during dose escalation phase:

ICI Agent	αPD-L1 (n=12)	αPD-L1+37.5mg αCTLA-4 (n=5)	αPD-L1+75mg αCTLA-4 (n=7)	Total (n=24)
Reported dose-limiting toxicities- no. (%)				
Any	0 (0%)	0 (0%)	0 (0%)*	0 (0%)*

Treatment-related adverse events occurring in ≥10% of patients:

Characteristic	αPD-L1 only (n= 12)	αPD-L1+ 37.5mg αCTLA-4 (n= 5)	αPD-L1+ 75mg αCTLA-4 (n= 21)	Total (n=38)
**Blood and lymphatic system disorders**				
Anaemia	5 (41.7%)	1 (20.0%)	6 (28.6%)	12 (31.6%)
Neutropenia	3 (25.0%)	1 (20.0%)	3 (14.3%)	7 (18.4%)
Thrombocytopenia	4 (33.3%)	1 (20.0%)	3 (14.3%)	8 (21.1%)
**Eye disorders**				
Vision blurred	2 (16.7%)	0 (0.0%)	2 (9.5%)	4 (10.5%)
**Gastrointestinal disorders**				
Abdominal discomfort	1 (8.3%)	2 (40.0%)	2 (9.5%)	5 (13.2%)
Abdominal pain	4 (33.3%)	1 (20.0%)	4 (19.0%)	9 (23.7%)
Colitis	1 (8.3%)	0 (0.0%)	3 (14.3%)	4 (10.5%)
Constipation	5 (41.7%)	4 (80.0%)	5 (23.8%)	14 (36.8%)
Diarrhoea	9 (75.0%)	2 (40.0%)	11 (52.4%)	22 (57.9%)
Dysphagia	5 (41.7%)	3 (60.0%)	6 (28.6%)	14 (36.8%)
Nausea	10 (83.3%)	5 (100.0%)	13 (61.9%)	28 (73.7%)
Vomiting	11 (91.7%)	4 (80.0%)	9 (42.9%)	24 (63.2%)
**General disorders and administration site conditions**				
Fatigue	10 (83.3%)	5 (100.0%)	14 (66.7%)	29 (76.3%)
Pyrexia	3 (25.0%)	2 (40.0%)	0 (0.0%)	5 (13.2%)
**Infections and infestations**				
Lower respiratory tract infection	1 (8.3%)	0 (0.0%)	4 (19.0%)	5 (13.2%)
Oral candidiasis	3 (25.0%)	0 (0.0%)	1 (4.8%)	4 (10.5%)
Upper respiratory tract infection	0 (0.0%)	1 (20.0%)	3 (14.3%)	4 (10.5%)

Characteristic	αPD-L1 only (n= 12)	αPD-L1+ 37.5mg αCTLA-4 (n= 5)	αPD-L1+ 75mg αCTLA-4 (n= 21)	Total (n=38)
**Investigations**				
Alanine aminotransferase increased	0 (0.0%)	0 (0.0%)	4 (19.0%)	4 (10.5%)
Amylase increased	3 (25.0%)	1 (20.0%)	2 (9.5%)	6 (15.8%)
Aspartate aminotransferase increased	0 (0.0%)	0 (0.0%)	5 (23.8%)	5 (13.2%)
Lipase increased	6 (50.0%)	0 (0.0%)	1 (4.8%)	7 (18.4%)
Weight decreased	2 (16.7%)	3 (60.0%)	4 (19.0%)	9 (23.7%)
**Metabolism and nutrition disorders**				
Decreased appetite	2 (16.7%)	3 (60.0%)	3 (14.3%)	8 (21.1%)
Hypokalaemia	2 (16.7%)	1 (20.0%)	1 (4.8%)	4 (10.5%)
**Musculoskeletal and connective tissue disorders**				
Back pain	3 (25.0%)	0 (0.0%)	2 (9.5%)	5 (13.2%)
**Neoplasms benign, malignant and unspecified (incl cysts and polyps)**				
Malignant neoplasm progression	2 (16.7%)	1 (20.0%)	4 (19.0%)	7 (18.4%)
**Nervous system disorders**				
Dysaesthesia	1 (8.3%)	0 (0.0%)	3 (14.3%)	4 (10.5%)
Dysgeusia	4 (33.3%)	0 (0.0%)	0 (0.0%)	4 (10.5%)
Headache	1 (8.3%)	1 (20.0%)	2 (9.5%)	4 (10.5%)
Neuropathy peripheral	3 (25.0%)	2 (40.0%)	6 (28.6%)	11 (28.9%)
Paraesthesia	3 (25.0%)	1 (20.0%)	3 (14.3%)	7 (18.4%)
Peripheral sensory neuropathy	4 (33.3%)	1 (20.0%)	5 (23.8%)	10 (26.3%)
**Psychiatric disorders**				
Insomnia	3 (25.0%)	1 (20.0%)	1 (4.8%)	5 (13.2%)
**Respiratory, thoracic and mediastinal disorders**				
Cough	5 (41.7%)	2 (40.0%)	4 (19.0%)	11 (28.9%)
Dyspnoea	2 (16.7%)	2 (40.0%)	4 (19.0%)	8 (21.1%)
**Skin and subcutaneous tissue disorders**				
Dry skin	2 (16.7%)	1 (20.0%)	1 (4.8%)	4 (10.5%)
Palmar-plantar erythrodysaesthesia syndrome	1 (8.3%)	0 (0.0%)	5 (23.8%)	6 (15.8%)
Pruritus	0 (0.0%)	0 (0.0%)	5 (23.8%)	5 (13.2%)
Rash	0 (0.0%)	1 (20.0%)	3 (14.3%)	4 (10.5%)

Table S2 | Treatment-emergent adverse events during the LUD2015-005 study (related to Table 1)
Top: A summary of all treatment-emergent adverse events (TEAEs) reported for the intent-to-treat (ITT) population of the inoperable LUD2015-005 trial cohorts. All TEAEs are shown, regardless of the degree of investigator-assessed likelihood that the TEAE was related to study drugs. Durvalumab-related TEAEs are defined as any degree of investigator-assessed likelihood of relationship to durvalumab. Severity of TEAEs was graded using Common Terminology Criteria for Adverse Events (CTCAE; v4.03)[7].
Middle: Reporting of dose-limiting toxicities (DLTs) in the dose escalation phase of the LUD2015-005 study. The pre-specified measurement window for this primary outcome was less than 10 weeks following first dose of medication. *1 of 14 patients in the extension phase of the αPD-L1+75mg αCTLA-4 cohort experienced an adverse event meeting DLT criteria.
Bottom: Frequency of individual TEAEs meeting a 10% cut-off across all patients in the ITT population are reported by their preferred term, which are categorized by system organ classes (bold).

CellPress

SUPPLEMENTAL REFERENCES

1. United States Department of Health and Human Services, Centers for Disease Control and Prevention (2018). National Program of Cancer Registries and Surveillance, Epidemiology, and End Results SEER*Stat Database: Incidence - SEER Research Data, Nov 2018 Sub. www.cdc.gov/cancer/uscs/public-use/.

2. Riaz, N., Havel, J.J., Makarov, V., Desrichard, A., Urba, W.J., Sims, J.S., Hodi, F.S., Martín-Algarra, S., Mandal, R., Sharfman, W.H., et al. (2017). Tumor and Microenvironment Evolution during Immunotherapy with Nivolumab. Cell *171*, 934-949.e15. https://doi.org/10.1016/j.cell.2017.09.028.

3. Hugo, W., Zaretsky, J.M., Sun, L., Song, C., Moreno, B.H., Hu-Lieskovan, S., Berent-Maoz, B., Pang, J., Chmielowski, B., Cherry, G., et al. (2016). Genomic and Transcriptomic Features of Response to Anti-PD-1 Therapy in Metastatic Melanoma. Cell *165*, 35–44. https://doi.org/10.1016/j.cell.2016.02.065.

4. Maag, J.L.V., Fisher, O.M., Levert-Mignon, A., Kaczorowski, D.C., Thomas, M.L., Hussey, D.J., Watson, D.I., Wettstein, A., Bobryshev, Y.V., Edwards, M., et al. (2017). Novel Aberrations Uncovered in Barrett's Esophagus and Esophageal Adenocarcinoma Using Whole Transcriptome Sequencing. Mol Cancer Res *15*, 1558–1569. https://doi.org/10.1158/1541-7786.MCR-17-0332.

5. Kim, S.T., Cristescu, R., Bass, A.J., Kim, K.-M., Odegaard, J.I., Kim, K., Liu, X.Q., Sher, X., Jung, H., Lee, M., et al. (2018). Comprehensive molecular characterization of clinical responses to PD-1 inhibition in metastatic gastric cancer. Nature Medicine *24*, 1449–1458. https://doi.org/10.1038/s41591-018-0101-z.

6. Cheong, J.-H., Wang, S.C., Park, S., Porembka, M.R., Christie, A.L., Kim, H., Kim, H.S., Zhu, H., Hyung, W.J., Noh, S.H., et al. (2022). Development and validation of a prognostic and predictive 32-gene signature for gastric cancer. Nat Commun *13*, 774. https://doi.org/10.1038/s41467-022-28437-y.

7. US Department of Health and Human Services (2010). Common Terminology Criteria for Adverse Events (CTCAE), Version 4.0.3. 80. https://evs.nci.nih.gov/ftp1/CTCAE/CTCAE_4.03/CTCAE_4.03_2010-06-14_QuickReference_8.5x11.pdf.
